# *SLAMR*, a synaptically targeted lncRNA, facilitates the consolidation of contextual fear memory

**DOI:** 10.21203/rs.3.rs-2489387/v1

**Published:** 2023-03-14

**Authors:** Isabel Espadas, Jenna Wingfield, Eddie Grinman, Ilika Ghosh, Kaushik Chanda, Yoshihisa Nakahata, Karl Bauer, Bindu Raveendra, Michael Kiebler, Ryohei Yasuda, Vidhya Rangaraju, Sathyanarayanan Puthanveettil

**Affiliations:** 1 Department of Neuroscience, The Herbert Wertheim UF Scripps Institute for Biomedical Innovation & Technology, Jupiter, FL, USA.; 2 Max Planck Florida Institute, Jupiter, FL, USA.; 3 Biomedical Center (BMC), Department for Cell Biology, Medical Faculty, Ludwig-Maximilians-University of Munich, 82152 Planegg-Martinsried, Germany.

**Keywords:** lncRNA, transport, KIF5C, plasticity, memory, hippocampus

## Abstract

LncRNAs are involved in critical processes for cell homeostasis and function. However, it remains largely unknown whether and how the transcriptional regulation of long noncoding RNAs results in activity-dependent changes at the synapse and facilitate formation of long-term memories. Here, we report the identification of a novel lncRNA, SLAMR, that becomes enriched in CA1- but not in CA3-hippocampal neurons upon contextual fear conditioning. SLAMR is transported to dendrites via the molecular motor KIF5C and recruited to the synapse in response to stimulation. Loss of function of SLAMR reduced dendritic complexity and impaired activity dependent changes in spine structural plasticity. Interestingly, gain of function of SLAMR enhanced dendritic complexity, and spine density through enhanced translation. Analyses of the SLAMR interactome revealed its association with CaMKIIα protein through a 220-nucleotide element and its modulation of CaMKIIα activity. Furthermore, loss-of-function of SLAMR in CA1 selectively impairs consolidation but neither acquisition, recall, nor extinction of fear memory and spatial memory. Together, these results establish a new mechanism for activity dependent changes at the synapse and consolidation of contextual fear.

## One sentence summary

Here we report identification and characterization of a novel lncRNA, SLAMR, that is regulated by experience and transported to stimulated synapses for mediating structural plasticity and fear memory consolidation.

## Introduction

Specific changes in transcription (Alberini et al., 1994; Fernandez-Albert et al., 2019; Marco et al., 2020), local translation (Martin et al., 1997; Van Driesche et al., 2018) and axonal transport (Puthanveettil et al., 2008; Guedes-Dias & Holzbaur, 2019; Swarnkar et al., 2021, Joseph et al., 2021) result in the formation of new synapses and the modification of preexisting ones (Miniaci et al., 2008; Bailey et al., 2015). These are well known mechanisms underlying the formation of long-term memories (LTM). However, we do not know what regulates these key steps and their spatial-temporal control. Particularly, we do not know how changes in the transcriptome of individual neurons might enable structural modifications at the synapse and in LTM.

The transcriptional changes associated with learning are very intricate. They rely on multiple components of the transcriptome undergoing unique changes in specific neuronal populations for LTM. Recent advances in next-generation sequencing technology have unraveled the complexity of the transcriptome and led to the discovery of new families of noncoding RNAs. Among them, long-noncoding RNAs (lncRNAs) are especially fascinating because of their participation in regulating translation in the cytoplasm and epigenetic changes in the nucleus suggesting their potential as key mediators of LTM (Li et al., 2018; Grinman et al., 2020; Cui et al., 2022). Importantly, an estimated 40% of these lncRNAs are specifically enriched in the brain (Derrien et al., 2012; Kaushik et al., 2013). Recent studies, including our own, have suggested that lncRNAs might be critically involved in essential neuronal functions such as synaptic remodeling and transmission, synaptogenesis, neurogenesis, and neuronal differentiation (Bernard et al., 2010; Mercer et al., 2010; Ng et al., 2013; Kour & Rath, 2017; Sarangdhar et al., 2017; Li et al., 2018; Raveendra et al., 2018; Keihani et al., 2019; Grinman et al, 2021; Cui et al., 2022). In addition, previous studies show that different brain regions implicated in memory, display unique lncRNAs profiles, such as the prefrontal cortex (PFC), amygdala, and hippocampus (Mercer et al., 2008; Kadakkuzha et al., 2015). Specifically, the hippocampus has shown a distinctive expression pattern of lncRNAs in different subregions of the tri-synaptic circuitry (Kadakkuzha et al., 2015). These specific patterns suggest that lncRNAs may play distinct roles in modulating neuronal functions during LTM. In addition, changes in patterns of expression of lncRNAs can be regulated in an activity-dependent manner (Barry et al., 2014, 2017; Li et al., 2018; Butler et al., 2019; Keihani et al., 2019; Grinman et al., 2021).

Despite these advances in our understanding of the neurobiology of lncRNAs, their roles in mediating signaling at the subcellular level and in modulating neuronal plasticity remain obscure. We, therefore, asked whether contextual fear conditioning (CFC), a form of associative learning resulting in robust and long-lasting fear memories, can induce the expression of lncRNAs in specific neuronal populations and whether they function in different types of LTM. We first carried out unbiased analyses of gene expression in CA1-hippocampal neurons to identify lncRNA changes induced by CFC and identified an enrichment of a novel lncRNA that we term “SLAMR” (*S*ynaptically *L*ocalized *A*ctivity *M*odulated lnc*R*NA) in dorsal CA1. SLAMR is mainly cytosolic and is transported to neuronal dendrites and into the spine compartment. Time-lapse quantitative imaging in combination with glutamate uncaging studies show that SLAMR is transported to dendrites through KIF5C, a molecular motor protein, and controls dendritic complexity and activity-dependent synaptic structural changes. We next identified a critical sequence element in SLAMR that is necessary for its transport and interaction with specific proteins. Importantly, we find that SLAMR expression changes modulate the activity of CaMKIIα in synaptoneurosomes. In addition, restricting SLAMR expression in the CA1 impairs the consolidation of CFC, whereas spatial memory remained intact. Taken together these results demonstrate that the novel lncRNA SLAMR plays a critical role in hippocampal-dependent associative LTM and the underlaying molecular, structural and functional processes.

## Results

### Contextual fear conditioning induces specific transcriptional changes in the coding and noncoding transcriptome of dorsal CA1

**Figure 1.**
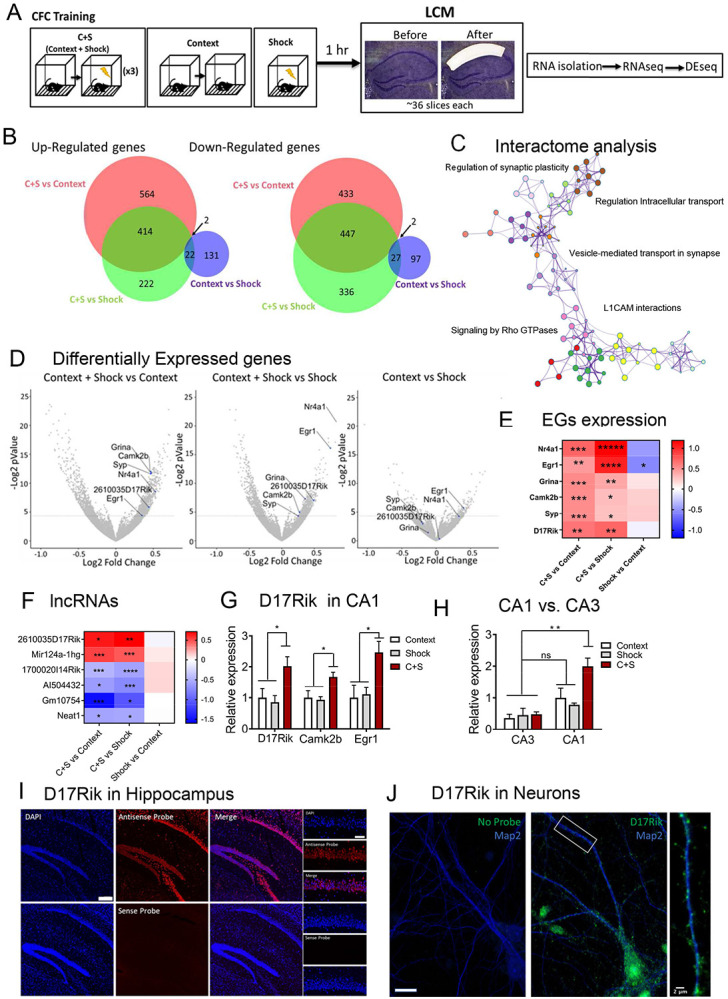
lncRNA D17Rik is enriched in dorsal CA1 after CFC. **A. Experimental Design.** 1hr after CFC training brains were isolated and fast frozen. After cryosectioning the dorsal hippocampus, the tissue was stained with an RNAse-free cresyl violet kit. CA1 dorsal area was dissected by LCM. Collected tissue was processed for RNA isolation using a trizol-chloroform protocol (250-400ng), prepared for RNAseq and the results were analyzed by the Bioconductor DEseq for “R”. **B. CFC induces transcriptional changes in dorsal CA1.** Proportional Venn diagrams derived from the DEseq analysis show important transcriptional changes in the experimental group (context + shock) compared to control groups (context alone and immediate shock) (p-value <0.05). **C. Significant changes in genes in CFC condition are related to early plasticity.** Cytoscape visualization of Metascape analysis indicates that most of the genes significantly up-or down-regulated in C+S condition compared to controls are grouped in a single cluster network related to early synaptic plasticity changes. **D. CFC is followed by an increase in the lncRNA D17Rik and plasticity-related genes.** DEseq results of dorsal CA1 represented by volcano-plots show a higher enrichment of the lncRNA D17Rik and plasticity-related genes in the context+shock group compared to context alone and immediate shock conditions (p-value<0.05). **E. EGs heat map.** Heat map represents log2Foldchange of EGs differentially expression in CFC. **F. lncRNAs heat map.** Heat map represents log2Foldchange of some of the most important lncRNAs regulated by the training in dorsal CA1. **G. CFC lncRNA D17Rik is significantly increased in dorsal CA1 after CFC.** RTqPCR results confirm a significant increase of D17Rik in dorsal CA1 (n=3-4) 1h after CFC training followed by an increase in synaptic plasticity-related genes. One-Way ANOVA, Multiple Comparisons Dunnett’s test C+S condition. Data is shown as MEAN±SEM. *p<0.05, **p<0.01, ***p<0.005, ****p<0.001. **H. D17Rik is not enriched in CA3 after CFC.** RTqPCR results show that the expression of D17Rik is enriched only in CA1 after CFC in the C+S, but not in CA3. There are no significant differences between CA1 and CA3 D17Rik expression in control groups (n=3-4). Two-way ANOVA + Tukey’s test. **p< 0.01. **I. Fluorescence in situ hybridization** (FISH) show that the lncRNA D17Rik is expressed in mouse hippocampus. High magnification details from pyramidal layer in CA1 indicate a mainly cytoplasmic subcellular localization of D17Rik. Confocal microscopy photomicrographs show D17Rik signal in red and DAPI signal from the nucleus in blue. Scale bars = 200 μm. **J. D17Rik localized in the Primary hippocampal neurons.** Fluorescence in situ hybridization (FISH) show that the lncRNA D17Rik is expressed in neurons in mouse primary hippocampal cultures. D17Rik (green) colocalizes with dendritic marker Map2(blue). Cell body scale bar=20μm. Dendrite inset scale bar=2μm.

In search of lncRNAs in dorsal CA1 neurons modulated by experience, we used CFC training, a behavioral paradigm for establishing robust LTM storage (Nakazawa et al., 2016). This behavioral paradigm provides the advantage of being able to independently analyze each memory phase. To identify lncRNAs differentially regulated during memory, 1 hour after CFC, we isolated the dorsal CA1 by laser capture microdissection (LCM) and extracted RNAs for total RNAseq. LCM isolated CA1 RNAs from shock alone (S) and context alone (C) were used as controls for identifying differentially expressed genes (DEGs) induced by experience (Figure 1A, Supplementary Figure S1A). The Venn diagrams shown in Figure 1B indicates the total number of genes, encompassing the coding and non-coding transcriptome, was significantly up-regulated or down-regulated based on a *p*-value<0.05 compared (~400 DEGs) with context alone and immediate shock (GSE214838) (Supplementary Table S1B). In contrast, the comparison between control conditions (context alone or immediate shock) identified ~100 DEGs. These results suggest that CFC induced significant and specific transcriptional changes in dorsal-CA1 neurons.

The CFC induced DEGs were mainly related to synaptic transmission and synaptic plasticity (Figure 1C). Interactome analysis using Metascape suggests that the enriched pathways are mainly related within a single cluster which includes: the regulation of synaptic plasticity, vesicle transport at the synapse, L1_CAM interactions and Rho GTPases signaling (p-adjusted <0.05, Figure 1C). We next searched for the presence of well-known mRNAs related to plasticity processes induced by neuronal activity to confirm the efficiency of the CFC training. We characterized the specific transcriptome profile in the dorsal CA1 for CFC training. The analysis of the genes that were upregulated in the C+S compared to both individual control groups, revealed several genes related to short- and long-term plasticity processes. Cellular component analysis indicated a significant enrichment in several synapse communication pathways like postsynaptic density, postsynaptic specialization, and neuron-to-neuron synapse (p-adjusted<0.0005; Supplementary Figure S1B and Table S1B. Biological processes analysis indicated a large enrichment in neuron specialization and synaptic plasticity, protein modification, and localization, among others (p-adjusted<0.05, Supplementary Figure S1C and Table S1C) suggesting an increase in different metabolic processes and plasticity changes in CA1 neurons induced by experience. Furthermore, the molecular function analysis showed regulation of transcriptional and translational processes induced by CFC in the dorsal CA1 with significant changes in genes related with mitochondrial activity. Specifically, genes involved in NADH activity (ex. Ndufa10, Ndufa11, Cbr1), ribonucleotide binding (ex. Ckmt1, Matk, Tubb5) (p-adjusted<0.05, Supplementary Figure S1D, and Table S1D) suggesting noteworthy changes in the activity of CA1 dorsal neurons specifically induced by CFC compared to either shock alone or context alone controls.

Additionally, DEseq analysis of RNAseq data (Figure 1D and E, Supplementary Tables S1D, and E) identified critical genes exclusively upregulated in CFC trained mice compared to controls. Some of these genes have also been found to be up-regulated in previous studies in the hippocampus of mice following CFC training or be involved in hippocampal LTP: for example, Egr-1 (Jones et al., 2001; Granado et al., 2008), the glutamate receptor Grina, Camk2β (Incontro et al., 2018; Wang et al., 2017), Nr4a1 (Chen et al., 2014; Oliveira et al., 2018) and SYP (Synaptophysin, Janz et al., 1999). Taken together, these results identified selective experience-dependent changes in the coding transcriptome of the CA1.

We next closely examined the noncoding RNAs identified by our DEseq analysis. In total, we identified 11 lncRNAs differentially expressed (DE) in the C+S condition compared to both control groups (Figure 1F, Supplementary Table S1F). Most of these lncRNAs have unknown functions, except for Neat1, a well-studied lncRNA which plays important roles in memory functions and stress responses in mice (Butler et al., 2019; Kukharsky et al., 2020). Neat1 is significantly downregulated in the C+S condition compared to context and shock alone (p<0.05). As previous studies demonstrated, Neat1 is known to suppress the immediate-early gene c-Fos whose activation is essential in early stages of memory acquisition (Butler et al., 2019).

The resulting DE lncRNAs were next classified into different subcategories based on their biotypes using the Ensemble annotation library (NCBIM37): sense_intronic, antisense, and long intergenic noncoding RNAs (lncRNAs), among others. Here, we identified 6 lncRNAs which showed significant changes in the C+S condition exclusively, indicating dynamic regulation of these lncRNAs related to hippocampal activity induced by CFC training in the dorsal-CA1 (Figure 1F). DEseq results showed that two of these genes (2610035D17Rik, Mir124a-1hg) were up-regulated (*p*-value<0.05) in the C+S condition within the dorsal CA1. Both lncRNAs are strong candidates for crucial regulators of neuronal plasticity process associated with memory. In fact, the lncRNA neuroLNC that is a rat homolog of Mir124a-1hg in mice is already described to be implicated in neurogenesis and presynaptic activity (Keihani et al., 2019).

### Discovery of a novel lncRNA in CA1 neurons modulated by CFC

Among the upregulated lncRNAs in CA1, we next focused on a previously undescribed lncRNA- 2610035D17Rik (D17Rik) as its expression is modulated in the CA1 by CFC. D17Rik is located in chromosome 11 and consists of 3 exons with non-coding potential indicated by phyloCSF and CPAT analysis (Wang et al., 2013) (Supplementary Figures S1E-G). Independent validation by qRT-PCR analysis confirmed that this gene is significantly up-regulated only in the C+S group and its upregulation is also accompanied by an increase in the expression of two plasticity related mRNAs, *CaMK2β* and *Egr-1* in the same samples (n= 3-4 per group. One-Way ANOVA, Dunnett’s test Figure 1G.), supporting the RNAseq findings. Interestingly, D17Rik expression in CA3 is not significantly enriched in any of the experimental groups (Figure 1H, Supplementary Table S1H) suggesting region-specific regulation of D17Rik expression in hippocampus. Together, these results demonstrate that D17Rik is an experience-dependent lncRNA specifically enriched in the dorsal CA1 hippocampal area following CFC training, suggesting a role in mediating contextual fear memory in the CA1.

We next examined the evolutionary conservation of D17Rik by searching for its orthologs. We found two D17Rik orthologs in humans and zebrafish, the LINC00673 and LOC110366352 (SlincR) respectively (Supplementary Figures S1H-J), (Kim et al., 2015; Li et al., 2017; Garcia et al., 2017). Tissue specificity of SlincR in zebrafish is not really known. The zebrafish transcriptome sequencing project (BioProject PRJEB1986) seems to indicate that this lncRNA is preferentially expressed in the head of adult males. Similarly, LINC00673 in humans seems to be well expressed in the brain according to the Illumina bodyMap2 transcriptome BioProject (PRJEB2445) and HPA RNA-seq normal tissues BioProject (PRJEB4337) in the NCBI databases. Interestingly, the loci for these three theoretical orthologs are conserved; especially regarding their position to other neighbor transcripts like Sox9 and Slc39a11, in these three species (Supplementary Figures S1H-J). In fact, the previously mentioned studies (Li et al., 2017; Garcia et al., 2017) suggested potential direct regulation of Sox9 mRNA by these lncRNAs due to the proximity (within 200 kb) of their promoters. As several lncRNAs are well known to have the ability to regulate other transcripts in a *cis*-manner, we decided to explore whether SOX9 could be a target of D17Rik (Supplementary Figure S1K). Our RNAseq data showed a significant reduction of Sox9 in the dorsal CA1 after CFC training exclusively in the C+S condition compared to both control groups (Supplementary Figure S1L). The log2foldchange data for D17Rik and Sox9, seems to indicate a negative correlation between them, suggesting a repression of the transcriptional processes (Supplementary Figure S1M). To further explore this regulation, we carried out RNAi mediated knockdown of D17Rik using antisense locked nucleic acid, Gapmer, oligonucleotides in *in vivo* and *in vitro* models and assessed the expression of Sox9. Gapmers have previously been successfully used to knockdown lncRNAs (Raveendra et al., 2018, Grinman et al., 2021). D17Rik silencing in our *in vitro* studies using primary hippocampal cell cultures did not result in significant changes in the expression of the genes in the D17Rik locus, including Sox9 (Supplementary Figure S1N). Consistently, in our *in vivo* studies silencing D17Rik in the dorsal CA1 of mice hippocampus also showed no changes in Sox9 when compared to the negative control. However, silencing D17Rik *in vivo* did show a significant reduction in the expression of the somatostatin receptor 2 (Sstr2) (n=7 both groups, *p<0.05, Unpaired t test; Supplementary Figure S1O).

To gain insight into the function of D17Rik, we next assessed its subcellular location by fluorescence *in situ* hybridization (FISH) and qRT-PCR analysis of cellular fractions. FISH imaging of D17Rik showed its expression in different hippocampal subareas (Figure 1I, Supplementary Figure S1P), enriched in the cytoplasm of pyramidal neurons (Figure 1J). To confirm this, we analyzed cytoplasmic and nuclear fractions from hippocampus and analyzed D17Rik’s expression by qRT-PCR. Using Actin as a cytoplasmic control for normalization and reference, we found that D17Rik is primarily expressed in the cytoplasm of the hippocampal neurons compared to the nucleus (n=3, *p<0.05, Student’s t test), while other well-known genes are equally distributed in both compartments (Map2), or specifically enriched in the nucleus (n=3 for both groups, Xist: *p<0.05; Gm9968: **p<0.0005, Student’s t test) (Supplementary Figure 1Q). These results confirm that D17Rik is enriched in the cytoplasm of pyramidal neurons. In addition, FISH analysis of D17Rik localization in cultured primary hippocampal neurons showed punctate distribution in dendrites (Figure 1J) indicating that D17Rik might be transported to dendrites.

### D17Rik displays dendritic transport in hippocampal neurons and is transported into dendritic spines

The dendritic localization of D17Rik suggests the possibility of a specific molecular motor mediated transport process. We sought to directly test this by visualizing dendritic transport of D17Rik. Based on is localization and modulation of its expression by experience, we named D17Rik lncRNA as SLAMR (*S*ynaptically *L*ocalized *A*ctivity *M*odulated lnc*R*NA).

**Figure 2.**
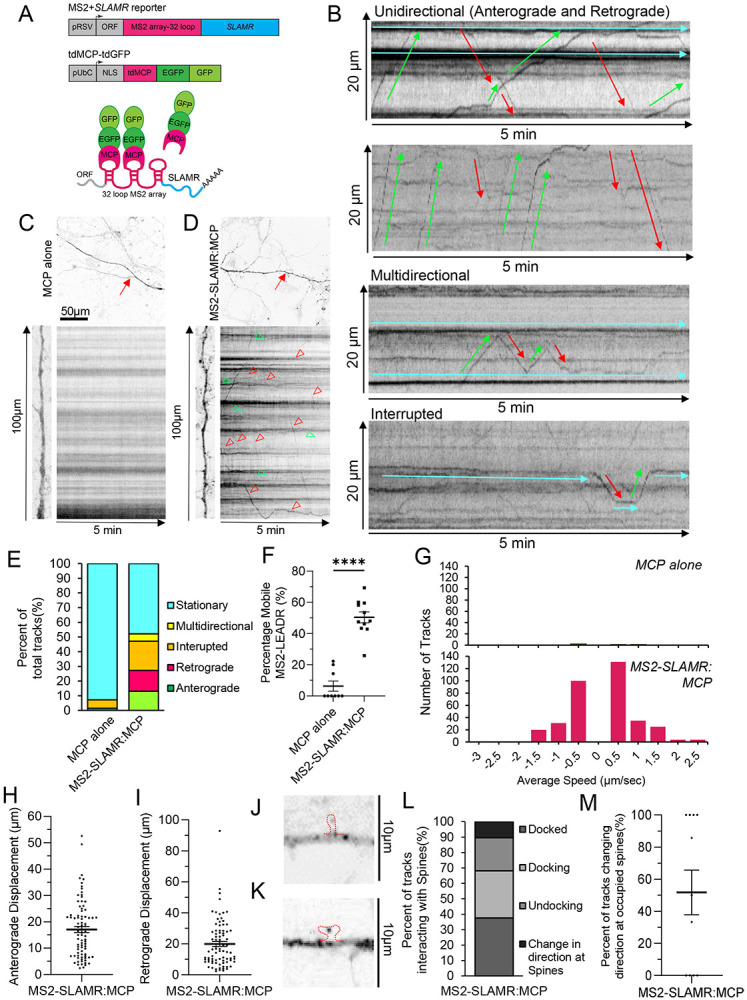
MS2-SLAMR displays directed dendritic transport in hippocampal neurons. **A.** Scheme of MS2-SLAMR reporter constructs and tdMCP-GFP expression cassettes (upper) and the MS2 system (lower). pRSV Rous sarcoma virus promoter, pUBC Ubiquitin C promoter, ORF open reading frame, NLS nuclear localization signal, tdMCP tandem MS2 coat protein. **B.** Representative kymographs illustrating differences in unidirectional MS2-SLAMR mRNA granule transport speed, displacement, and directionality, as well as multidirectional transport and interrupted. A few highlighted anterograde and retrograde transport and stationary tracks are indicated in green or red or blue arrowheads and lines, respectively. **C, D.** Top and left: dendritic branch which the kymograph and movies were taken from. Red arrows point to the specific branch. Right: representative kymograph from neuron transfected mcp alone (**C**) or MS2-SLAMR (**D**) and MCP. Soma is orientated towards the bottom; distal dendrite end is orientated towards the top. Distance progresses along the vertical axis, time progresses along the horizontal axis. Red arrowheads point to retrograde tracks. Green arrows point towards anterograde tracks. **E.** Quantification of relative transport dynamics of MS2 only and MS2-SLAMR reporter mRNA in 5 -min time-series acquisitions. **F.** Percent of mobile MCP alone or MS2-SLAMR puncta. Individual values shown. Mean±SEM. Student’s t-test. ****p-value<0.0001 **G.** Distribution of speeds of MS2-SLAMR and MCP alone in primary hippocampal neurons. **H.** Anterograde displacement of MS2-SLAMR. Individual tracks shown. Mean±SEM. **I.** Retrograde displacement of MS2-SLAMR. Individual tracks shown. Mean±SEM. **J.** MS2-SLAMR:MCP granule in a thin spine. Red outline of spine based on red channel RcAMP. **K.** MS2-SLAMR:MCP granule in a mushroom spine. Red outline of spine based on red channel RcAMP. **L.** Distribution of MS2-SLAMR dynamics in relation to dendritic spines (labeled with PSD95-mCherry). **M.** Percent of MS2-SLAMR granules that change direction at a dendritic spine already occupied by MS2-SLAMR. Results from individual neurons shown. Mean±SEM.

For live imaging of RNA transport using the MS2-MCP system (Bertrand et al., 1998; Hocine et al., 2013; Bauer et al., 2017, 2019; Tuttuci et al., 2018), we prepared plasmid constructs to express MCP and full-length SLAMR tagged with MS2 binding sites (MBS). For these experiments, we used primary rat neuronal hippocampal cultures that were co-transfected with the coat protein tdMCP-GFP and MS2-SLAMR reporter RNA (Figure 2A). GFP signal from MCP constructs is usually localized in the nucleus, but after MS2-SLAMR tandem synthesis, MCP binds MS2 loops in the cytoplasm allowing the visualization of SLAMR transport following GFP positive particles traveling through the neuron. FISH labeling targeting SLAMR demonstrated that MS2-MCP constructs co-localized in both the cytoplasm and dendrites (Supplementary Figure S2A), confirming that we reliably detected the lncRNA SLAMR, allowing the visualization of its transport in living neurons.

Next, we investigated the dynamics of SLAMR transport through spinning-disc microscopy and imaging analysis of single neurons expressing the MS2 system. We recorded each neuron for 5 minutes at ~1 fps (frames per second) and analyzed the trafficking of single RNA granules. Kymographs of dendritic regions were generated at a distance of >10 μm from the soma and single trajectories identified (Figures 2 B-D, Supplementary Figures S2 B-C, Supplementary Movies S1-4). This experiment revealed different RNA transport patterns, even for the same granules, in a single recording (anterograde, retrograde, multidirectional, and interrupted). These transport patterns are similar to those found for mRNAs in previous studies (Bauer et al., 2019) following the sushi-belt model. This suggests that mRNA granules travel through dendrites with a highly dynamic multidirectional behavior. The analysis of the frequencies of these types of transport found that less than 50% of the RNA particles remain stationary during the acquisition period (Figure 2E). This is significantly lower than granules in the control condition (MCP alone) where more than 90% remain stationary (Figure 2E). Furthermore, more than 50% of granules traversed the dendrites in a highly dynamic manner, including multidirectional, interrupted, retrograde and anterograde movements (MCP alone n=9 neurons, MS2-SLAMR:MCP n=11 neurons; ****p<0.0001, Student’s t-test; Figures 2E and F) in a 5-min time span of acquisition. While MS2-SLAMR and MCP alone granules on average, displayed similar anterograde and retrograde velocities to MCP alone (Anterograde: MCP alone=0.56±0.17μm/s, MS2-SLAMR:MCP=0.56±0.04μm/s) and (Retrograde: MCP alone=0.37±0.12μm/s, MS2-SLAMR:MCP=0.49±0.03μm/s) there were only 4 total tracks to measure in the MCP alone condition compared to 352 tracks of MS2-SLAMR:MCP (n=9 dendrites per condition, Student’s t-test, Figure 2G). Due to the extremely low number of tracks in the MCP alone condition preventing us from making meaningful statical comparisons with MS2-SLAMR:MCP, moving forward we only focused on analyzing MS2-SLAMR dynamics. MS2-SLAMR showed varied anterograde and retrograde displacement (n=9 neurons, Student’s t-test *p<0.05, Figures 2H and I). These results demonstrate that SLAMR is actively transported along the dendrites in a similar manner to previously reported mRNAs required for local translation of proteins required for plasticity processes (Bauer et al., 2019; Tuttuci et al., 2018).

Interestingly, we also observed that MS2-SLAMR could enter into a variety of dendritic spine compartments. Specifically, we noticed that MS2-SLAMR:MCP granules could localize within thin and mushroom spines (Figures 2J and K). To further character MS2-SLAMR’s behavior in relation to dendritic spines we imaged MS2-SLAMR concurrently with PSD95-mCherry to ensure we were observing its transport specifically in dendrites. We found that of MS2-SLAMR RNA granules frequently dock at, undock, or changed direction at dendritic spines labeled with PSD95 (Figure 2L, Supplementary Figures S2D-E, Supplementary Movies S5-6). Curiously, over 50% of the time MS2-SLAMR changed direction at a spine that was already occupied by an MS2-SLAMR granule (n=11 dendrites, Figure 2M). This observation suggests that there may be a mechanism determining whether the neuron already has ‘enough’ SLAMR at a spine and thus needs to direct it to an unoccupied spine.

### Mechanism of SLAMR dendritic transport and discovery of a molecular motor-dependent regulation

**Figure 3.**
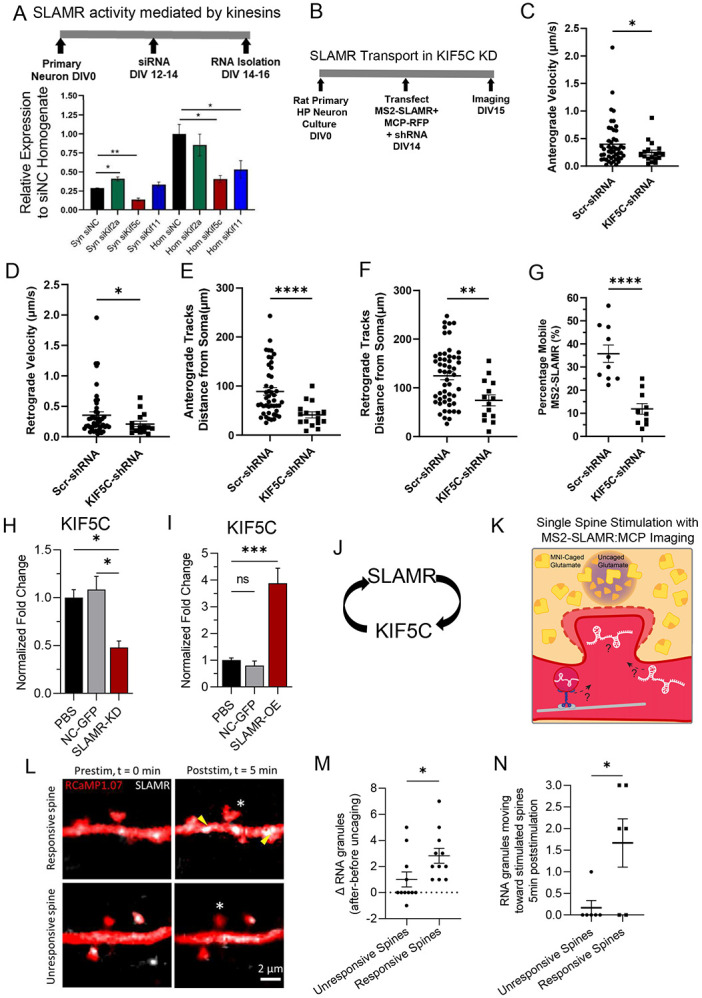
lncRNA SLAMR and kinesin KIF5C reciprocally regulate each other. **A.** Top: Experimental timeline. Bottom: RT-qPCR results show changes in SLAMR RNA levels in total homogenates and synaptic fractions of primary neurons after kinesin silencing. *p<0.05, **p<0.005. One-way ANOVA followed by Dunnett’s test. **B.** Experimental timeline for KIF5C KD and MS2-SLAMR: MCP-RFP dynamics analysis. **C-D.** Anterograde and retrograde velocity of MS2-SLAMR in KIF5C knockdown neurons is decreased compared to scrambled control knockdown. Student’s t-test. *p<0.05, Error bars=SEM. N=10 neurons for both conditions. Individual tracks shown. Mean±SEM. **E-F.** The distance from the soma that anterograde (E) and retrograde tracks (F) of MS2-SLAMR begin their transport is much closer to the soma in KIF5C knockdown neurons compared to scrambled control knockdown. Student’s t-test. **p<0.005, ****p<0.0001. Error bars=SEM. N=10 neurons for both conditions. **G.** The percent of mobile MS2-SLAMR: MCP-RFP granules is reduced in KIF5C knockdown neurons compared to scrambled control knockdown. Student’s t-test. ****p<0.0001, Error bars=SEM. N=10 neurons for both conditions. **H.** RT-qPCR results show decreased Kif5c mRNA abundance in neuronal cultures transduced by lentivirus containing shSLAMR compared to NC-GFP lentivirus or treatment with PBS. Student’s t-test *p-value<0.01, **p-value<0.05, ***p-value<0.005. Error bars=SEM. **I.** RT-qPCR results show increased Kif5c mRNA abundance in neuronal cultures transduced by lentivirus containing OE(over-expressed)-SLAMR compared to NC-GFP or treatment with PBS. Student’s t-test *p-value<0.01, **p-value<0.05, ***p-value<0.005. Error bars=SEM. **J.** Model of the reciprocal regulation of SLAMR and KIF5C. **K.** Diagram illustrating single spine stimulation DIV18-21 neurons were transfected with RcAMP1.07 (red) and MS2-SLAMR:MCP (white). 16-22hrs later these neurons were images and individual spines were stimulated with 30 pulses of 2 photon excitation to uncage MNI-Caged glutamate and evaluate transport dynamics of SLAMR to dendritic spines. **L.** Single frames from spine stimulation experiment prestimulation and 5 minutes after stimulation showing an example of an unresponsive spine which did not increase in volume and a responsive spine which did grow during local glutamate uncaging. Red=RcAMP, White puncta= MS2-SLAMR:MCP granules. White asterisk indicates stimulated spine. **M.** Results of examining the change in the number of RNA granules (MS2-SLAMR:MCP) before stimulation to 5min after stimulation in a 5μm dendritic region of the stimulated spine. Student’s t-test *p-value<0.05. Error bars=SEM. **N.** Results of examining the change number of RNA granules (MS2-SLAMR:MCP) moving toward the stimulated spine within 5min after stimulation in a 25μm region of the stimulated spine. Student’s t-test *p-value<0.05. Error bars=SEM.

As the dynamics of MS2-SLAMR suggest a motor-dependent transport, our next objective was to investigate which kinesins might be involved in SLAMR transport. We first selected three kinesins to test that participate in dendritic transport: KIF2A, KIF5C, and KIF11 (Twelvetrees et al., 2019; Grinman et al. 2021; Swarnkar et al. 2018, 2021). RT-qPCR results from total homogenates and synaptoneurosomes from primary neuronal hippocampal cultures, where these kinesins were silenced show that SLAMR expression significantly changes when transport by KIF2A, KIF5C, and KIF11 is individually disrupted in hippocampal neurons (n=3 per group, *p<0.05, **p<0.005. One-way ANOVA followed by Dunnett’s test, Figure 3A). Specifically, silencing of KIF2A increased SLAMR abundance in the synaptic fractions, suggesting that it acts as a negative regulator of SLAMR transport to the synapse. Silencing KIF5C decreased SLAMR in the homogenate and synaptic fraction, suggesting that it positively regulates the global abundance of SLAMR and its targeting to synapses. While silencing KIF11 only decreased SLAMR in the total homogenate, indicating that it is correlated with SLAMR expression but unlikely impacts its localization to synapses. Furthermore, overexpression of KIF5C resulted in an increase in the expression of SLAMR in homogenates as well as in synaptoneurosome fractions (n=3, p<0.05, student’s t-test; Supplementary Table S3A). Taken together, these results suggested the possibility that KIF5C might mediate dendritic transport of SLAMR. To further investigate this possibility, we examined the dynamics of MS2-SLAMR in KIF5C knock-down (KD) neurons.

Briefly, primary rat hippocampal neurons were transfected with MS2-SLAMR:MCP-RFP and either a scrambled (Scr-shRNA) control or KIF5C-shRNA construct, which has been previously established to lead to a significant decrease in KIF5C abundance in hippocampal neurons (Swarnkar et al. 2018, 2021) (Figure 3B). We then proceeded with spinning-disc microscopy of neurons co-expressing the SLAMR-MS2:MCP-RFP system and the indicated shRNA (resulting in eGFP expression). We recorded each neuron for 5 minutes at ~1 fps. For this analysis, we focused on 100μm dendritic regions where we observed robust trafficking of single RNA granules >10μm away from the soma and generated kymographs (Supplementary Figures S3A-D, Supplementary Movies S7-10). Loss-of-function of KIF5C significantly reduced the anterograde and retrograde velocities of MS2-SLAMR RNA granules compared to the Scr-shRNA control (n=10 per condition, Student’s t test *p<0.05, Figures 3C-D, Supplementary Tables S3C-D). Additionally, KIF5C loss-of-function significantly reduced the distance at which the anterograde and retrograde MS2-SLAMR granules begin their transport away from the soma compared to the Scr-shRNA control (n=10 per condition, **p<0.005, ****p<0.0001, Student’s t test; Figure 3E-F). Lastly, KIF5C knockdown significantly decreased the percentage of mobile MS2-SLAMR granules to 11.9±2.3% compared to 35.7±3.8 % mobile particles in the Scr-shRNA control (n=10 per condition, ****p<0.0001, Student’s t test, Figure 3G). Taken together, these results demonstrate that SLAMR depends on KIF5C for transport into dendrites.

As KIF5C demonstrated to be crucial for SLAMR function, we consider the possibility of a reciprocally regulation between these two elements. To elucidate this, we assessed the possibility of whether the expression of KIF5C is modulated by SLAMR. Therefore, we studied the effect of both the loss- and gain-of-function of SLAMR on KIF5C abundance in hippocampal neurons. Specifically, we treated primary hippocampal neurons with PBS or transduced them with lentiviral particles expressing negative control (NC-GFP), silencing (shSLAMR), or overexpression (OE-SLAMR) constructs for SLAMR. After extracting RNA from these cultures, and examining SLAMR expression to confirm knockdown and overexpression (Supplementary Figures S3E,F), we then examined the abundance of KIF5C. Interestingly, loss-of-function of SLAMR diminished the level of KIF5C to 0.48 ± 0.07 fold compared to the NC-GFP control (n=4 per condition; one-way ANOVA followed by Tukey’s multiple comparison test; Figure 3H) while gain-of-function of SLAMR increased KIF5C by 3.877 ± 0.56 fold compared to NC-GFP control (n=4 for PBS, and NC-GFP, n=3 for OE-SLAMR; one-way ANOVA followed by Tukey’s multiple comparison test; Figure 3I). Together, these results suggest that SLAMR and KIF5C reciprocally regulate each other’s expression in primary hippocampal neurons (Figure 3J).

### SLAMR is recruited to stimulated spines

As SLAMR expression increases in the hippocampus following CFC, is actively transported to dendrites and is localized to spine compartments, this led us to consider the possibility that SLAMR might be recruited to spines in response to stimulation for activity dependent structural changes. Supporting this idea, a few other RNAs have been demonstrated to increase in dendrites and dendritic spines following stimulation (Grinman et. al 2021, Bauer et al. 2019). Thus, we next examined whether local glutamate stimulation impacts SLAMR localization to spines in hippocampal neurons. To test recruitment of SLAMR to activated spines, we used two-photon (2p) excitation glutamate uncaging to stimulate individual dendritic spines in neurons expressing MS2-SLAMR:MCP and RcAMP (volume marker) (Figure 3K). We then compared the recruitment of SLAMR to spines which were responsive to the stimulation by showing and increased spine head width, to those that did not (n=11 unresponsive spines, n=11 responsive spines; *p<0.05, ***p<0.001, ****p<0.0001, two-way ANOVA followed by Sidak’s multiple comparison test; Figure 3L, Supplementary Figure S3G). We found that the responsive spines had an increase of ~3 RNA granules within a 5μm dendritic region of the stimulated spine compared to a modest recruitment of ~1 RNA granule at nonresponsive spines, 5 minutes after stimulation (Nonresponsive n=11 spines, Responsive n=11 spines, Student’s t test, *p-value<0.05; Figure 3L,M). We next examined the direction that SLAMR was moving in a 25 μm dendritic region of the stimulated spines following local glutamate uncaging. Here, we found that in the 5min following stimulation, the responsive spines showed ~2 RNA granule moving toward the stimulated spine, while the non-responsive spines showed ~0.2 RNA granules moving toward the stimulated spine (Nonresponsive n=6 spines, Responsive n=6 spines; *p-value<0.05 Student’s t test; Figure 3N, Supplementary Figures S3H,I, Supplementary Movies S11,12). Together, these results demonstrate the selective recruitment of SLAMR to spines exhibiting morphological plasticity.

### The expression of SLAMR is required for dendritic arborization and activity–dependent structural changes

**Figure 4.**
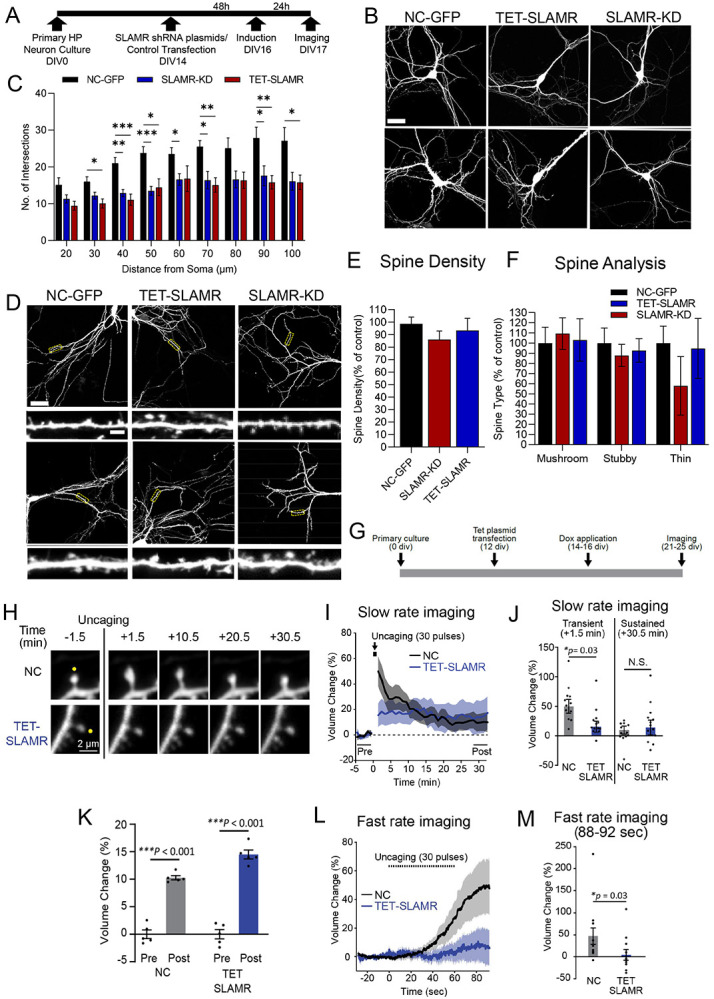
SLAMR facilitates dendritic arborization and transient structural plasticity. **A.** Experimental timeline for morphology studies. **B.** Confocal projection images show soma in the center to depict the dendritic arbor. Scale Bar=40μm. **C.** Quantification of dendritic morphology changes using Sholl analysis of intersections per 10-μm step size. Changes compared between SLAMR constitutive and inducible (TET) KD and NC-GFP(negative control). Two-way ANOVA followed by Tukey’s post hoc test. Error bars represent SEM. *p<0.05, **p<0.005, ***p<0.0005. **D.** Confocal projection images showing area analyzed for spine morphology and enlarged image in the inset for spine details. Cell Body Scale Bar=40μm. Dendrite inset Scale bar=2μm. **E-F.** SLAMR KD did not induce significant changes in the total number of spines nor their different subtypes. Error bars represent SEM. One-way ANOVA. **G.** Experimental timeline of hippocampal primary culture, inducible shRNA plasmid transfection, and doxycycline treatment for two-photon live cell imaging with glutamate uncaging. **H.** Representative fluorescence images of SLAMR shRNA-EGFP (TERT-SLAMR) and scrambled shRNA-EGFP (NC-GFP) before and after glutamate uncaging pulse (30 pulses, 0.5 Hz). Scale bar, 2 μm. **H-K.** Glutamate uncaging analysis of spine changes in volume shows a significant decrease in TET-SLAMR condition compared with NC-GFP control at transient phase. Two-way or One-way ANOVA, followed by Tukey’s multiple comparisons test. Error bars represent SEM. *p<0.05, ***p<0.001. **L-M.** Fast rate analysis of the spine volume during glutamate uncaging. Error bars represent SEM. Mann-Whitney U test. *p<0.05.

After we established that SLAMR is transported throughout dendrites and is recruited to spines in an activity-dependent manner, we next sought to examine if SLAMR functions more broadly in regulating the morphology of the dendritic arbor and spines. To do so, we carried out loss of function experiments by shRNA KD of SLAMR to determine the necessity of its expression for synapse function. We used an shRNA plasmid to constitutively silence the expression of SLAMR and alternatively, another one under a doxycycline-inducible promoter (TET) for temporal control of SLAMR KD. A plasmid expressing a scrambled sequence was used as negative control (NC). All plasmids also express eGFP to visualize transfected neurons and assess morphological changes (Figure 4A,B). Sholl analysis of intersections shows that both plasmids for SLAMR silencing (shRNA and TET-ON condition) induce a significant decrease in dendritic arborization of the neurons transfected compared to the NC (NC-GFP n=9, SLAMR-KD n=10, TET-SLAMR n=11; *p<0.05, **p<0.005, ***p<0.0005, Two-way ANOVA followed by Tukey’s post hoc test; Figures 4B-C). However, we did not find any significant changes in spine number and morphology in the cells transfected compared to the NC condition (Figures 4D-F). Since the loss of function has resulted in a significant decrease in overall arborization, these results indicate that SLAMR expression facilitates dendritic morphology and the total number of synapses.

Though the loss-of-function experiments did not result in a decrease in number of spines per 100 μm dendritic length, we were curious about the functionality of the spines as we observed recruitment of SLAMR to stimulated spines that went through structural plasticity. Therefore, we first investigated the effect of SLAMR KD during the induction of structural long-term potentiation (sLTP) in single dendritic spines by local glutamate uncaging which is a model of functional synaptic plasticity and learning (Matsuzaki et al.,2004).

In addition, we employed shRNA plasmids targeting SLAMR (TET-SLAMR) or NC-GFP (Figure 4G) and assessed activity dependent changes in spine morphology. Structural long-term potentiation (sLTP) was induced in single dendritic spines of primary cultured hippocampal neurons by local glutamate uncaging with 2p excitation. This uncaging stimulus induced a large transient volume increase (49.9±11.8% at +1.5 min after the first uncaging) at the stimulated spine expressing the control shRNA (n = 12 spines from 4 cells) (Figure 4H-J). However, the spine enlargement was significantly suppressed (15.6±9.3% at +1.5 min after the first uncaging) at a transient phase in neurons that inhibited endogenous SLAMR (n = 13 spines from 5 cells; p= 0.03, two-way ANOVA followed by Tukey’s post hoc test; Figures 4 I-J). Interestingly, a sustained sLTP (+28.5-32.5 min after the first uncaging) was induced and maintained not only in the control group (10.3±0.4%) (p<0.001; one-way ANOVA followed by Tukey’s post hoc test) but also in SLAMR KD group (14.5±0.8%) (p< 0.001, two-way ANOVA followed by Tukey’s post hoc test; Figures 4I-J). The degree of sustained spine growth was comparable between each group (NC 9.8±6.9% and SLAMR KD 14.0±12.6% at +30.5 min) (p = 0.97, two-way ANOVA followed by Tukey’s post hoc test; Figures 4K). To examine whether endogenous SLAMR is responsible for the sensitivity of postsynaptic structural remodeling, a fast rate of image acquisition was performed during glutamate uncaging. Spine growth was induced during a train of glutamate uncaging and kept increasing immediately after the stimulation in the control group (48.8±18.8% between 88-92 sec after the 1st uncaging) (n = 12 spines from 4 cells; Figures 4L and M). However, the initiation of spine growth was not observed during uncaging and the spine enlargement was significantly attenuated in the SLAMR -KD group (6.7±12.3% between 88-92 sec after the first uncaging) (n = 11 spines from 4 cells; p = 0.03, Mann-Whitney’s U test; Figures 4L-M). Taken together, we conclude from these results that SLAMR is involved in the transient structural plasticity of spines but not critical for sustained sLTP.

### Gain-of-function of SLAMR enhances dendritic arborization and spine density

**Figure 5.**
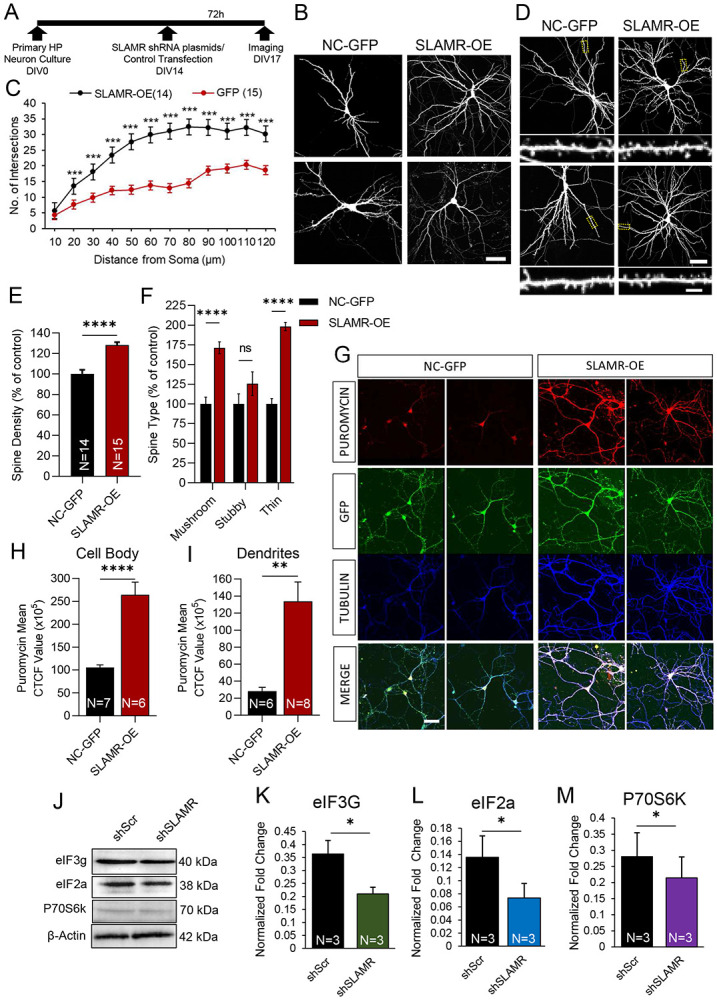
Gain-of-function of SLAMR results in enhanced dendritic arborization, spine density, and local protein synthesis. **A.** Experimental timeline for morphology studies. **B.** Confocal projection images show soma in the center to depict dendritic arbor. Scale Bar=40μm. **C.** Quantification of dendritic morphology changes using Sholl analysis of intersections per 10-μm step size. Changes compared between NC-GFP and SLAMROE (overexpression). Two-way ANOVA followed by Tukey’s post hoc test. Error bars represent SEM. *p<0.05, **p<0.005, ***p<0.0005. **D.** Confocal projection images showing area analyzed for spine morphology and digitally enlarged image in the inset for spine details. Cell Body Scale Bar=40μm. Dendrite inset Scale bar=2μm. **E-F.** SLAMR-OE induced significant increases in the spine density (student’s t-test) and percent of thin and mushroom spines (One-way ANOVA) Error bars represent SEM. **G.** Confocal projection images of NC-GFP and SLAMR-OE transfected images stained for Puromycin, GFP, α-tubulin, and the merged image. Scale Bar=40μm. **H-I.** Quantification of G based on Corrected Total Cell Fluorescence (CTCF). SLAMR-OE induced significant increases in puromycin staining (student’s t-test) in the cell body (**G**) and in dendrites (**I**). Error bars represent SEM. **J.** Loss-of-function of SLAMR results in diminished components of local translation machinery. Western blot analysis of β-actin, P70S6K, eIF2a, eIF3g, isolated from primary hippocampal neurons transduced by either shScr or SLAMR lentivirus synaptoneurosomes. **K-M.** Quantification of (**J**) normalized to β-actin (student’s t-test) Error bars represent SEM. *p<0.05.

We next asked whether enhancing the expression of SLAMR would produce an enhancement in dendritic arborization and spine density. Therefore, we carried out gain of function experiments by transfecting primary hippocampal neurons with a plasmid to express full length SLAMR (SLAMR -OE) under the control of a CMV promoter. We also include an empty backbone plasmid as negative control (NC-GFP) (Figure 5A). Sholl analysis of intersections shows that compared to NC-GFP, SLAMR-OE induces a significant increase in the number of branches (NC-GFP, n=15, SLAMR-OE, n=14; *p<0.05, **p<0.005, ***p<0.0005, Two-way ANOVA followed by Tukey’s post hoc test; Figures 5B-C). Additionally, we found an increase in spine density and in the percentage of thin and mushroom spines in neurons transfected with SLAMR-OE compared to the NC-GFP condition (NC-GFP, n=15, SLAMR -OE, n=17; *p<0.05, **p<0.005, ***p<0.0005, student’s t test (E) and Two-way ANOVA followed by Šídák's multiple comparisons test (F); Figures 5D-F). These results, together with the loss of function experiments, indicate that SLAMR is an essential mediator of dendritic arborization and spine morphology.

The observation that SLAMR-OE was sufficient to induce an increase in arborization as well as the reciprocal regulation of SLAMR and KIF5C, suggested the possibility of a translational change in hippocampal neurons. Previously, we reported that KIF5C-OE produced an increase in translation in hippocampal neurons (Swarnkar et al., 2021). Thus, we investigated whether changes in SLAMR levels affected local translation. First, we carried out puromycin labeling of newly synthesized proteins in NC-GFP and SLAMR-OE neurons, followed by immunostaining. SLAMR-OE resulted in increased staining of puromycin-labeled proteins in the cell body (SLAMR-OE=1643875.089 ± 257055.9475 CTCF, n= 6), compared to the NC-GFP control (NC-GFP=1055403.278 ± 57712.63243 CTCF, n=4) (****p<0.0001, unpaired Student’s t test; Figures 5G-H). Such changes were also observed in dendrites (SLAMR -OE=1339079 ± 226821.7 CTCF, n = 8) when compared to NC-GFP control (NC-GFP=581139.3 ± 45647.45 CTCF, n = 6) (**p < 0.001, unpaired two-tailed Student’s t test; Figures 5G, I).

To better understand the role of SLAMR in modulating neuronal translation, we next evaluated whether loss-of-function of SLAMR diminishes local translation at the synapse. We isolated synaptoneurosomes from primary hippocampal neurons transduced with lentivirus containing scrambled shRNA (shScr) or SLAMR shRNA (shSLAMR) and probed for the presence of the translation machinery components. We first validated the isolation of synaptoneurosomes by probing for synaptic marker glutamate receptor (GluR2) and synaptophysin in total homogenate, cytosolic, and synaptoneurosome fractions. We found GluR2 and Synaptophysin highly enriched in the synaptoneurosome fraction compared to the total homogenate and cytosolic fraction (n=3 per condition; ***p<0.001, ****p<0.0001, One-way ANOVA followed by Tukey’s multiple comparison’s test; Supplementary Figures S4 A-C). Western blot analysis of synaptoneurosomes relative to NC-GFP, showed shSLAMR decreased markers for protein synthesis such as, eIF3g (shSLAMR =0.21 ± 0.03, shScr=0.36 ± 0.05, n=3), eIF2a (shSLAMR =0.07 ± 0.02, shScr=0.14 ± 0.03, n=3), and P70S6K (shSLAMR =0.21 ± 0.06, shScr= 0.28 ± 0.07, n=3) (*p < 0.05; unpaired two-tailed Student’s t test; Figures 5 J-M). This result suggests that SLAMR is a mediator of translation in hippocampal neurons.

### Unbiased analyses of SLAMR interactions identify specific lncRNAs, miRNAs, mRNAs and proteins

**Figure 6.**
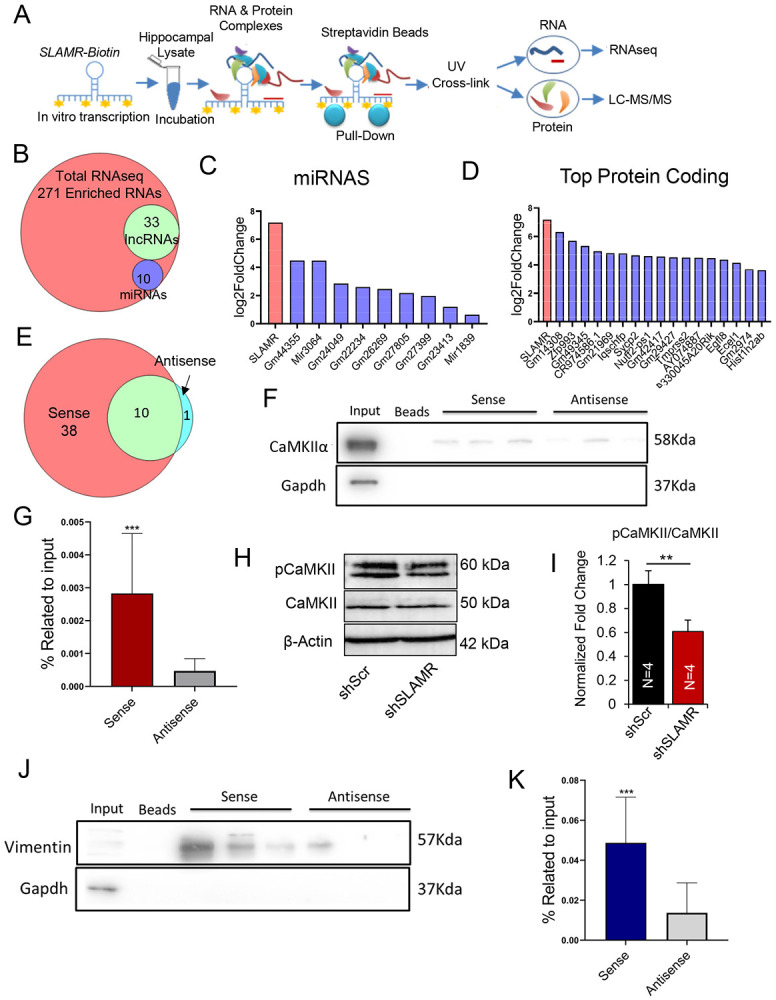
lncRNA SLAMR interacts with several miRNAs and proteins. **A.** Experimental design using pull-down purification assay with SLAMR full-length biotinylated probes. **B.** Venn-diagram indicates the number of total RNAs enriched in the sense condition, number of miRNAs and lncRNAs. **C.** miRNAs identified after small RNA sequencing interacting with SLAMR. **D.** Log2Fold change of the top 20 coding and non-coding genes after pulldown total RNAseq. **E.** Venn-diagram represents the number of proteins identified by LC-MS/MS for each experimental condition. **F.** Representative CaMKIIα western blot. Three lanes of replicative pulldowns using sense and antisense SLAMR are shown. **G.** Bar diagram of the results obtained after WB analysis of CaMKIIα based on the percentage of input. N=12, 4 experiments x 3 replicates (***p<0.0005, Unpaired t test). **H.** Western blot analysis of β-actin, phosphorylated CaMKII(p-CaMKII) and CaMKII isolated from primary hippocampal neurons transduced by either shScr or shSLAMR lentivirus synaptoneurosomes. **I.** Quantification of (**H**) normalized to β-actin, relative to shScr. (student’s t-test) **p<0.005. Error bars represent SEM. **J.** Representative Vimentin immunoblot. Three lanes of replicative pulldowns using sense and antisense SLAMR are shown. **K.** Bar diagram represents the results of WB analysis for Vimentin over the input percentage. N= 12, 4 independent pull-downs with 3 replicates each (***p<0.0005, Unpaired t test).

To gain further insight into the mechanism of SLAMR function, we isolated SLAMR-containing complexes from intact hippocampus and analyzed the associated RNA and protein components by total RNAseq, small RNAseq, and proteomics. Briefly, we used a biotinylated probe containing the full-length sense sequence of SLAMR (Figure 6A), or a biotinylated antisense strand of SLAMR as a negative control (Supplementary Figure S5A) to pull-down SLAMR complexes. RT-qPCR analysis of isolated complexes indicated a significant enrichment of SLAMR, supporting the efficiency of SLAMR pulldown (Supplementary Figure S5B). Silver staining following SDS-PAGE analysis of eluted complexes revealed proteins between 60 to 40 KDa (Supplementary Figure S5C). The complexes purified after pull-down were processed for both, RNA and protein isolation. The RNA samples were submitted for both total RNAseq and small RNAseq; proteins were analyzed using liquid chromatography mass spectrometry (LC-MS/MS). This resulted in the identification of 271 coding and non-coding transcripts enriched in the sense condition (p-value<0.05) as potential candidates (Figure 6B). From these transcripts, 10 miRNAs were identified (Figure 6C and Supplementary Table S5C) most of them with unknown functions (ex. Gm44355, Gm24049, Gm22234), except for mir3064 and mir1839 that were previously described to be involved in post-transcriptional and translational regulation (Bai et al., 2017). We next used Metascape to analyze the list of mRNAs significantly enriched in the sense condition (Figure 6D, Supplementary Table S5D). Results from this analysis showed that most of the mRNAs that interact with SLAMR are involved in functions like the electron transport chain, regulation of ion transmembrane transport, transsynaptic signaling or positive regulation of excitatory postsynaptic potential, indicating a crucial role of this lncRNA in mitochondrial function and synaptic transmission.

LC-MS/MS analysis identified 38 unique proteins in the sense condition (Figure 6E). Those were found to be involved in diverse functions including mitochondrial function (ex. ATP synthase subunit β), transcription regulation (ex. Histone H2A) or cytoskeleton neurofilaments (see Supplementary Table S6E). We focused on two specific proteins that were validated by western blot (WB): the Calcium-calmodulin protein kinase alpha (CaMKIIα) and the intermediate filament Vimentin. LC-MS/MS experiment identified 5 unique peptides for CaMKIIα (Supplementary Figure S5D) that were also validated by WB through 4 independent pull-down experiments with 3 replicates each (n=12 total; ***p<0.0005, unpaired Student’s t test; Figures 6 F-G). It is well known that CaMKIIα plays an essential role in excitatory synaptic transmission and plasticity and that phosphorylation of CaMKII T282 is a prerequisite for this function (Liu & Jones, 1996; Incontro et al., 2018). Then, we were curious if SLAMR had a role in the phosphorylation of CaMKII at the synapse. Therefore, we isolated synaptoneurosomes from primary neuronal cultures transfected with control shRNA (shScr) or SLAMR shRNA (shSLAMR) and probed for CaMKII and phosphorylated (T282) CaMKII (Figure 6H). Reducing SLAMR in neuronal cultures led to a significant reduction in the ratio of phosphorylated to total CaMKII in synaptoneurosomes (n=4 per condition; **p<0.01, Student’s unpaired t test; Figure 6I, Supplementary Table S5I).

During the SLAMR-Biotin pulldown we were also able to identify 20 unique peptides for Vimentin by LC-MS/MS (Supplementary Figure S5E). The immunoblot analysis for Vimentin validated its enrichment in the sense condition in several different independent experiments (4x3 replicates, n=12; ***p<0.0005, unpaired Student’s t test; Figures 6J and K). These intriguing findings suggest a possible role of SLAMR in plasticity processes and hippocampal-dependent memory. Vimentin is a key factor in CNS cell differentiation and neurite development (Boyne et al., 1996; Wilhelmsson et al., 2019). These two proteins are involved in different processes directly and indirectly related to learning and memory, and the study of their interactions with SLAMR is crucial to understanding how this lncRNA is required for hippocampal-mediated memory consolidation.

### A 220-nucleotide fragment of SLAMR is sufficient to interact with CaMKIIα and Vimentin

**Figure 7.**
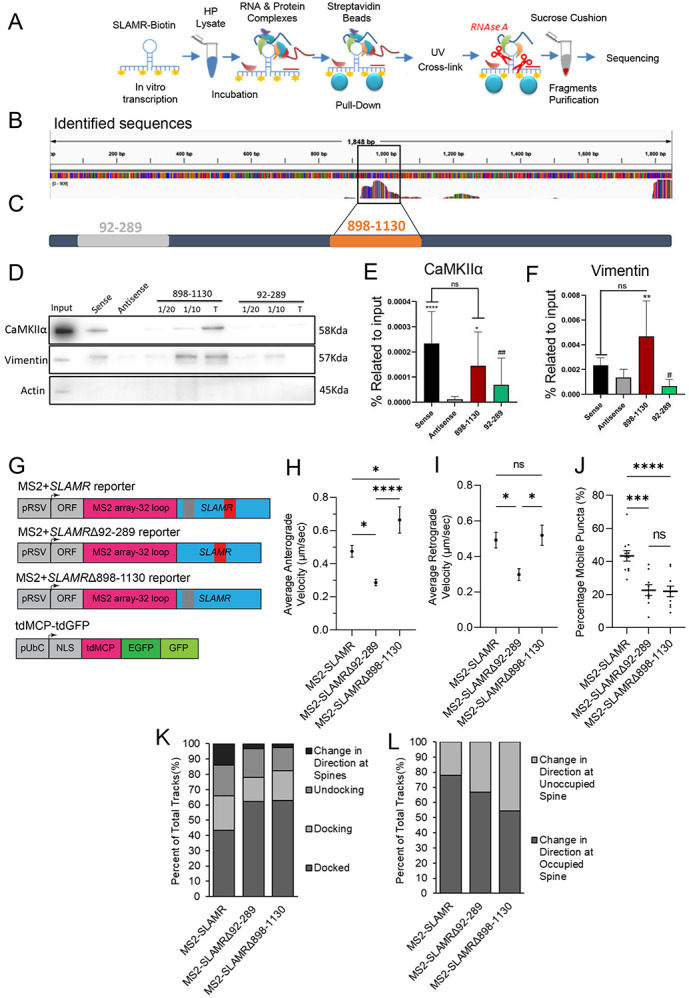
CaMKIIα and Vimentin interact with a specific fragment of the lncRNA SLAMR. **A.** Schematic of approach to identify protein-protected fragments of SLAMR using a pull-down strategy followed by RNAse A treatment and sucrose cushion purification. Obtained fragments were then submitted for sequencing. **B.** Mapping of seq-Reads of the mouse genome using Salmon and IGV software for visualization. **C.** Scheme of cloned SLAMR fragments used to design specific biotinylated PD probes. **D.** Representative immunoblot images stained for CaMKIIα, Vimentin and Actin from WB with PD samples. **E-F.** Plots indicate the results of WB quantification for CaMKIIα (Independent experiments, N=11-12, vs Antisense ****p<0.0001, *p<0.05; vs sense ##p=0.0032. One-way ANOVA + Tukey’s test) and Vimentin (Independent experiments, N=5-6, **p<0.01. One-way ANOVA + Tukey’s test) **G.** Schematic of MS2-SLAMR truncated constructs. **H-J**. Quantifications of Supplementary Figure 7A-F Kymographs. **H.** Deletion of the alternative (92-289) fragment decreased the anterograde speed while deletion of Vimentin/CaMKII(898-1130) binding site increased the speed of MS2-SLAMR:MCP granules compared to the full-length MS2-SLAMR. Error bars represent SEM. ****p<0.0001, *p<0.05. **I.** Deletion of the alternative (92-289) fragment decreased the retrograde speed while deletion of the vimentin/CaMKII(898-1130) binding site led to no change compared to the full-length MS2-SLAMR. *p<0.05. **J.** Deletion of both the control (92-289) and the vimentin/CaMKII (898-1130) binding site reduced the percent mobile MS2-SLAMR:MCP granules compared to the full-length MS2-SLAMR. ***p<0.001, ****p<0.0001. Error bars=SEM. **K-L.** Quantifications of Supplementary Figure 7D-F Kymographs. **K** Distribution of how MS2-SLAMR, MS2-SLAMRΔ92-289 and MS2-SLAMRΔ898-1130 interact with dendritic spines. **L.** Both the deletions of the alternative fragment and vimentin/CaMKII binding sites decrease the percentage of times that MS2-SLAMR turned around at dendritic spines already occupied by MS2-SLAMR.

We next sought to determine which regions of SLAMR interact with protein components of its interactome. Therefore, we carried out RNAse protection assays to identify interacting (protected) region within SLAMR. Following RNAse-A digestion and sucrose cushion centrifugation to isolate protected fragments for RNAseq analysis, (Figure 7A) (Carrieri et al., 2012), we identified multiple RNAse-protected fragments from SLAMR, one of which showed a major peak spanning a segment of around 220nt in length in the middle of its sequence (Figure 7B). Additionally, one other fragment showed a low number of reads and/or were incomplete, similar to another one at the 3’ end. We next asked whether the 220 nt fragment is sufficient to interact with either CamKIIα or Vimentin. We therefore prepared a biotin labelled sense probe for the major peak, and another probe from a different region of SLAMR corresponding to a minor peak (Figure 7C) and carried out pulldown experiments. In addition, we also included a SLAMR full-length sense probe as a positive control and an antisense probe as a negative control. Western blot analysis confirmed that the fragment identified by RNAseq between 898-1130nt was sufficient to pull-down both proteins. CaMKIIα (n=11-12; vs Antisense ****p<0.0001, *p<0.05; vs sense ##p=0.0032, One-way ANOVA + Tukey’s test; Figures 7 D,E) and Vimentin (n=5-6; **p<0.01. One-way ANOVA + Tukey’s test; Figures 7 D,F) showed enrichment with the 898-1130nt probe compared with the antisense condition. The results indicate that the 898-1130 region of SLAMR is important for the formation of RNA-associated protein (RAP) complexes.

RAP complexes can mediate the interaction between lncRNAs and the motor proteins which transport them (Chennathukuzhi et al., 2003). Thus, we sought to explore whether this 220nt region (from 898 to −1130nt) of SLAMR participates in SLAMR trafficking. For this experiment we designed two new MS2-SLAMR constructs; one with an alternative fragment deleted, MS2-SLAMRΔ92-289, and one with the CaMKIIα/Vimentin binding region deleted, MS2-SLAMRΔ898-1130 (Figure 7G). We proceeded with imaging as previously described for MS2-SLAMR: tdGFP-MCP (Supplementary Figures S6A-F, Supplementary Movies S13-18). Curiously, the 92-289nt deletion decreased both the anterograde and retrograde MS2-SLAMR velocity compared with the intact SLAMR, while the 898-1130nt deletion significantly increased the anterograde but not the retrograde velocity of MS2-SLAMR (n=9,11,9 neurons respectively MS2-SLAMR, MS2-SLAMRΔ92-289, MS2-SLAMRΔ898-1130; *p<0.05, **p<0.005. One-way ANOVA followed by Tukey’s test, Figures 7H-I, Supplementary Figures S6A-F and Movies S13-18). Both the 92-289nt and 898-1130nt deletions significantly decreased the percentage of mobile MS2-SLAMR granules to 23.9±3.6% and 24.3±3.8%, respectively, compared with the intact SLAMR which had 46.9±4.7% mobile granules, (n=11,9,10 for MS2-SLAMR, MS2-SLAMRΔ92-289, and MS2-SLAMRΔ898-1130, respectively; **p<0.005. One-way ANOVA followed by Tukey’s test; Figure 7J). Furthermore, both these deletions decreased the percentage of MS2-SLAMR mobile granules interacting with dendritic spines and the number of particles that changed directions at spines already occupied with MS2-SLAMR from ~78%, to ~69% for MS2-SLAMRΔ92-289 and ~55% of MS2-SLAMRΔ898-1130 RNA granules (Figures 7K-L, Supplementary Figures S6G-L, and Movies S19-24). Together, these results demonstrate that SLAMR integrity is critical for proper transport within dendrites.

### Expression of SLAMR in CA1 is involved in the consolidation, but not required for acquisition, extinction, and recall of contextual fear conditioning

Functional manipulation of SLAMR, activity-dependent synaptic structural changes, quantitative analysis of trafficking into spines, and analysis of its interactome suggested a key role for SLAMR in mediating LTM. Therefore, we assessed the effect of loss-of-function of SLAMR by RNAi mediated knockdown of SLAMR using Gapmer oligonucleotides in CA1 in multiple memory processes such as acquisition, consolidation, extinction and extinction recall of contextual fear memory. We first designed three different Gapmer antisense oligonucleotides (Supplementary Figures S7A-B) that were tested in primary hippocampal cell cultures. Hippocampal cell cultures were transfected independently with different Gapmers that either target SLAMR or a negative control (NC) sequence that does not have a specific target in mice (75’ AACACGTCTATACGC 3’, Raveendra et al, 2018) (Supplementary Figure S7B). Total RNA was isolated 42 and 72 hrs after transfection (Supplementary Figure S7C). The qRT-PCR analysis of these RNAs indicated that the levels of SLAMR 72hrs after transfection with Gapmer_1 were significantly reduced compared to the negative control (Gapmer1=0,286±0,051, NC=1±0,150, n=4 per condition; ***p-value<0.005, One-Way ANOVA + Dunnett’s test; Supplementary Figure S7C) with higher efficiency than Gapmers 2 and 3. Furthermore, before performing the behavioral experiments, we tested Gapmer_1 from the previous experiments (named SLAMR_Gapmer for the following experiments) *in vivo* to evaluate its efficiency. Mice received bilateral stereotaxic infusions in the dorsal CA1 hippocampal area of SLAMR_Gapmer using the JetSI delivery method. 72hrs after the surgery mice were sacrificed and the area around the infusion was dissected from the hippocampus to isolate the RNA. The qRT-PCR results indicated that SLAMR_Gapmer was able to induce a 40% reduction of the expression of SLAMR in the hippocampus (NC=1±0.142, SLAMR_Gapmer=0.630±0.059, n=7 per condition; *p-value<0.05, Student’s *t* test; Supplementary Figure S7D.).

**Figure 8.**
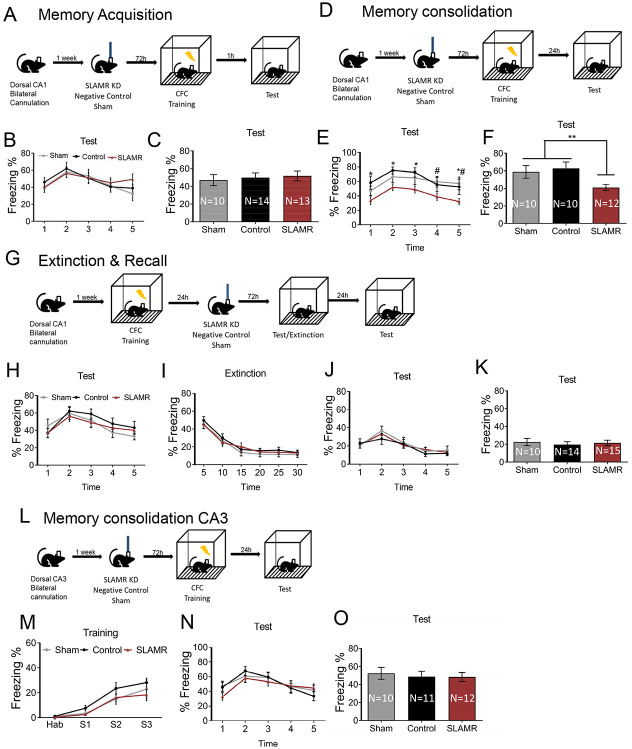
SLAMR is implicated in contextual fear memory consolidation. **A.** For the acquisition experiment, mice were implanted with bilateral cannulas in the dorsal CA1 and then divided into three groups (sham, NC and SLAMR_Gapmer) and infused with their corresponding agent 72hrs before the CFC training. **B-C.** 1hr after training these mice were placed again in the same context for a 5 min test. There were no differences between groups in the early retention test. **D.** SLAMR was KD in dorsal CA1 of mice implanted with bilateral cannulas 72h before the training and tested 24h later in the same context. **E-F.** The long-term memory test was carried-out 24h after the training and shows a significant reduction in the percentage of freezing time on those mice where the SLAMR was KD by the Gapmers compared to controls. Two-way ANOVA followed by Tukey’s test. ^#^p<0.05, *p<0.05, **p<0.01. Data is represented by MEAN±SEM. **G.** Experimental design where SLAMR was KD after memory consolidation. **H.** SLAMR KD 24hr after finishing the training does not affect the expression of fear during the long-term memory test. **I.** The percentage of freezing during the 30 mins of the extinction process does not show any differences between groups. **J-K.** The Recall test was performed 24 hrs after the extinction shows an effective reduction in the expression of freezing in all the groups. **L.** Experimental design for SLAMR manipulation in CA3. **M.** Plots show a similar percentage of freezing expression between groups during the training as well as (**N-O)** 24h after the training the long-term memory test was performed.

After validating the functionality of SLAMR_Gapmer we evaluated the role of SLAMR in early stages of memory acquisition. For this experiment, bilateral cannula targeting the CA1 were implanted into mice. One week after the surgery, mice were divided into 3 groups which received a single infusion of their corresponding Gapmer into the CA1 dorsal area: SLAMR_Gapmer, NC_Gapmer or Sham (non-infused). 72hrs after the infusion, all mice were trained in CFC and 1 hour after the training they were tested in the same context for 5 minutes without any shock delivery to evaluate the acquisition of conditioned fear response (Figure 8A). The results indicated that silencing the expression of SLAMR in dorsal-CA1 did not affect the expression of fear during the training (Supplementary Figure S7E,H) and did not alter the acquisition of the conditioned response (SLAMR=51.53±5.55; Control=49.87±5.27; Sham=47±6.2; Figures 8B-C). All groups (Sham, NC, and SLAMR_Gapmer) showed similar percentages of freezing values in both training and test sessions.

To evaluate the role of SLAMR in the consolidation of CFC memory, we designed the following experimental procedure similar to the previous one (Figure 8D): 72hrs after the infusion, all mice were trained in CFC and 24 hours after the training they were tested in the same context for 5 minutes without any shock delivery to evaluate the memory consolidation. Results showed no differences between groups in the expression of fear during the training (Supplementary Figure S7F,I). However, we found a significant reduction in the percentage of total freezing time during the 24hr test in those mice that received the Gapmer which silenced the expression of SLAMR. Whereas sham and negative controls showed similar values during the test session (SLAMR=41.016±3.579; Control=62.754±7,385; Sham=58.895±7,162; ^#^*p<0.05, **p<0.01, Two-way ANOVA followed by Tukey’s test; Figures 8E and F). Together, these results indicate that the reduction in SLAMR expression in dorsal CA1 impaired the long-term memory consolidation of the CFC, while the expression of fear during the training remained intact.

Additionally, when SLAMR was knocked-down 24hrs after training (Figure 8G, Supplementary Figure S7G,J) when the memory had already consolidated, this did not interfere with memory expression (Figure 8H). Also, extinction training performed 72hr after SLAMR inactivation did not show any differences in the percentage of freezing values between groups (Figure 8I). Furthermore, the recall test performed 24hrs after extinction training, verified the extinction efficiency through a high reduction in the percentage of freezing levels in all the groups. Recall test values are similar between treatments indicating that SLAMR activation in dorsal-CA1 is not necessary for this process (SLAMR=21.53±3.04; Control=19.60±3,26; Sham=22.55±4.08; Figures 8J-K).

Previous studies demonstrated that activity-dependent transcriptional changes induced by CFC training take place not only in CA1 but also in the CA3 hippocampal subregion (Impey et al., 1998). To verify this, we also performed a behavioral study silencing the expression of SLAMR into dorsal-CA3 of the hippocampus following the same procedure for the CA1 experiments (Figure 8L; Supplementary Figure S7K). In this case, data showed no differences between any of these groups in the fear expression during the training (Figure 8M), as well as in the percentage of freezing time during the test for memory consolidation (Figures 8N-O). All the groups included in this study yielded similar values. These results indicate that SLAMR is not induced by CFC training and is not necessary for memory consolidation in the dorsal-CA3 hippocampal subregion. In summary, these results indicate that SLAMR seems to be critical specifically for the memory consolidation processes whereas the expression of fear, acquisition, extinction and recall functions remains intact after reducing SLAMR levels in dorsal CA1 or CA3 hippocampal areas.

### SLAMR expression in dorsal CA1 does not impact spatial learning and memory.

**Figure 9.**
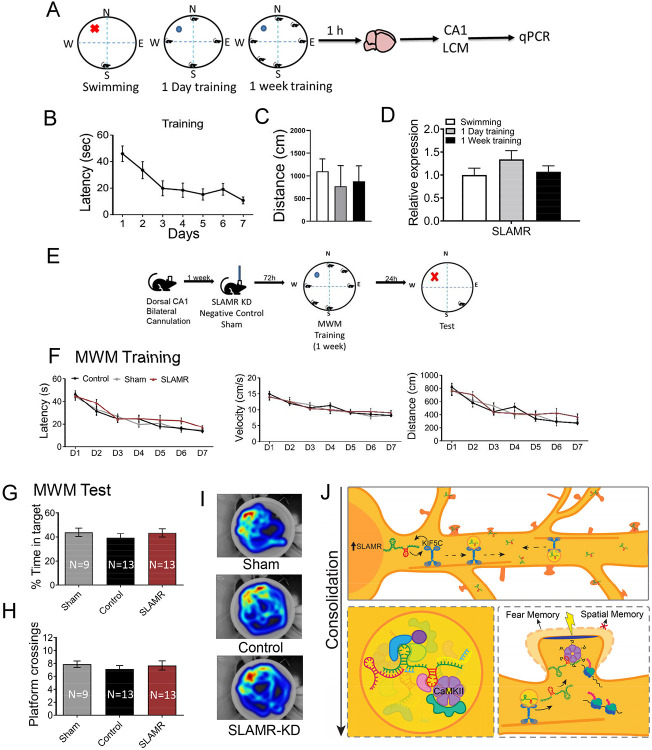
SLAMR is not enriched in dorsal CA1 after MWM training as well as not required for spatial learning and memory. **A.** Experimental design. **B.** Latency of mice in MWM during 1 week of training. **C.** Distance during the first trial or a single swimming session. **D.** RT-qPCR results show that lncRNA SLAMR is not increased in dorsal CA1 1 hr after finishing a single session of swimming exercise, 1 day or 1 week of training in MWM (n=4 per group). **E.** Schematic representation of SLAMR genetic manipulation for MWM study. **F.** Latency values did not show any differences between groups during the learning and long-term memory test. Also, no differences in distance and velocity values indicated behavioral changes induced by the genetic manipulation of SLAMR. **G-H.** Test results in percentage of time spent in target quadrant and platform crossings show no differences between groups for consolidation of spatial memory. Data shown as MEAN±SEM. **I.** Representative heat maps indicated normal exploratory behavior during the test session for all three conditions. **J.** Model of SLAMR during consolidation. Top panel: Learning increases SLAMR expression which reciprocally regulates KIF5C expression. KIF5C transports and deposits SLAMR throughout the dendrite and in spines. Bottom panel left: Model of a vesicle containing SLAMR and identified interactor CaMKII, along with numerous other proteins, lncRNAs, microRNAs, and mRNAs as suggested by the pulldown experiments. Red hairpin indicates binding region to CaMKII/Vimentin. Bottom panel right: Stimulated dendritic spine showing structural plasticity, the active recruitment of SLAMR, phosphorylation of CaMKII and increased local protein synthesis. SLAMR participates in this role in fear memory but not spatial memory.

It is well known that the dorsal CA1 of the hippocampus plays an essential role in spatial learning and memory processes (Moser et al., 1993, 1995). To determine whether SLAMR is implicated in other hippocampal-dependent behaviors, we performed the Morris Water Maze (MWM) assay to evaluate spatial hippocampal-dependent functions. Mice were divided into three different groups and trained in the MWM test for a week a single day or just exposed to a session of swimming without a platform to discard any influence induced by the novel environment or the exercise (Figures 9A and B). The graph in Figure 9C indicates that exploratory behavior during 1 week of training group, 1-day training group, or the swimming session was similar between groups. There were no differences at the basal level between different experimental groups. Then, 1 hr after finishing their respective trainings mice were sacrificed and the brains were isolated to dissect the CA1 dorsal area by LCM. The RNA obtained was reverse transcribed and qRT-PCR was performed to measure SLAMR expression. In this case, the qRT-PCR experiments did not show any significant differences between groups in dorsal CA1 in any of the three different conditions, indicating that SLAMR expression is not increased in this hippocampal subregion by MWM training (1 week=1,07±0.13; 1 day=1.34±0.19; Swimming=1±0.15; Figure 9D).

To confirm this observation, a second experiment was carried out manipulating the expression of SLAMR in dorsal-CA1 following a similar procedure from previous experiments of this study. As the experimental design indicates (Figure 9E), mice were cannulated to target dorsal-CA1 (Supplementary Figure S8A). A week after the surgery mice were treated with: SLAMR_Gapmer, NC_Gapmer, or Sham (non-infused). 72hrs after the infusion, mice were trained in the MWM for 7 days, with 4 trials each from randomly assigned starting positions. Then, 24 hrs after finishing the training, a long-term memory test was performed to evaluate the memory consolidation by removing the platform and using a novel starting position. During the training, none of the groups show significant changes between them in latency, distance, or velocity (Figure 9F). These results indicate that spatial learning, motor responses and/or motivation are all intact after cannula implantation and genetic manipulation in this brain region. Similarly, mice spent similar amounts of time in the target quadrant and number of crossings over the previous platform position (Figures 9 G-I), revealing that SLAMR genetic inactivation in dorsal CA1 does not affect spatial memory consolidation in this behavioral paradigm.

## Discussion

It is well known that specific transcriptional changes are required for many phases of LTM including consolidation, recall, or even for extinction processes (Flexner et al., 1963; Montarolo et al., 1986; Kida et al., 2002; Vianna et al., 2003; Lee et al., 2004; Berger-Sweeney et al., 2006; Bianchi et al., 2014; Alberini & Kandel, 2014). Previous studies indicate that lncRNAs are important components of transcriptional changes relevant for learning and LTM (Maag et al., 2015; Barry et al., 2017; Tan et al., 2017; Li et al., 2018; Butler et al., 2019; Raveendra et al., 2018; Grinman et al., 2021). However, little is known about lncRNAs localized to dendrites and their mechanism of function at the synapse. Here we describe the identification and detailed characterization of a previously undescribed lncRNA that we termed SLAMR in hippocampal neurons.

### SLAMR functions as a “master” regulator of structural plasticity

Having the proper levels of arborization and the ability to regulate structural and synaptic plasticity is critical for neurons to function in learning and LTM. Studies from our lab and other reveal that lncRNAs have important roles in regulating dendritic arborization and spine density and plasticity. Loss-of-function of Gm12371, a nuclear enriched lncRNA, resulted in the reduction in dendritic arborization, spine density and morphology (Raveendra et al., 2018) whereas ADEPTR loss of function produced reductions in spine density and morphology without altering dendritic arborization (Grinman et al., 2021). Furthermore, loss-of-function of BC1 increased spine density and size yet decreased dendritic arbor and structural plasticity (Briz et al., 2017), and loss-of-function of LoNA increased spine density (Li et al., 2018) Importantly, glutamate uncaging followed by two-photon imaging of dendritic spines show that ADEPTR deficient spines failed to undergo transient changes in spine morphology (Grinman et al., 2021). Interestingly, loss-of function of SLAMR produced significant decrease in dendritic arborization, although spine density and morphology of remaining dendritic spines were not altered. However, single spine stimulation and timelapse imaging of morphology suggest that the remaining spines (likely due to the reduction in arborization) are also deficient in undergoing activity-dependent structural changes. Taken together, these results suggest that key features of neuronal architecture could be modulated by the expression of specific lncRNAs.

While the loss-of-function of some lncRNAs resulted in specific morphological deficits, the consequences of gain-of-function of lncRNAs are much less understood. Here, we found that overexpression of SLAMR was sufficient to produce enhancements in dendritic arborization, spine density, and morphology. Further, we found that this enhancement is due to an increase in global translation produced by enhancing the expression of key modulators of translation such as eIF2 alpha and S6 Kinase. We recently showed that overexpression of molecular motor KIF5C produced global enhancements in translation (Swarnkar et al., 2021). Therefore, we examined whether the expression of KIF5C and SLAMR might be linked. We find that loss-of-function of KIF5C decreased the abundance of SLAMR in synaptoneurosomes whereas its overexpression produced an overall increase in SLAMR. Conversely, the loss-of-function of SLAMR resulted in reduced levels of KIF5C whereas its overexpression resulted in an increase in KIF5C levels. This surprising reciprocal regulation suggests that SLAMR overexpression results in the activation of KIF5C expression and function resulting in an increase in translation leading to morphological changes.

### SLAMR is transported into dendritic spines through the molecular motor KIF5C

While dendritic localization of populations of mRNAs and a few lncRNAs has been described (Muslimov et al., 2002; Moccia et al., 2003; Moroz et al., 2006; Puthanveettil et al., 2013; Cajigas et al., 2012; Bauer et al., 2019; Fonkeu et al., 2019; Grinman et al., 2021), the mechanism by which these RNAs are transported is much less understood. Several studies have demonstrated that kinesins play a crucial role in RNA transport (Kanai et al., 2004; Hirokawa et al., 2005; Puthanveettil et al.,2013, Swarnkar et al., 2021). Nonetheless, while there are hundreds of RNAs shown to be localized to hippocampal dendrites, we do not know which kinesins mediates their transport and how they do so.

We have recently shown that KIF5C is associated with ~700 RNAs and likely mediates their dendritic localization, becoming a potential candidate for lncRNAs transport. In addition, our earlier study on the dendritically localized ADEPTR lncRNA find that its localization depends on KIF2A, a KIF that was not previously known to mediate RNA localization (Grinman et al., 2021). We followed a similar approach to identify KIFs that mediates dendritic localization of SLAMR. qRT-PCR analysis of synaptoneurosomes prepared from hippocampal neurons in which different KIFs were knocked down using siRNAs suggested that KIF5C likely mediate SLAMR transport to dendrites. Taken together these results suggests that kinesins are responsible for dendritically targeting lncRNAs.

To gain deep insight into dendritic transport of SLAMR, we established quantitative analysis of transport of SLAMR by expressing MCP and MS2-tagged SLAMR in hippocampal neurons. Apart from exhibiting the transport kinetics of KIF mediated transport of SLAMR, we also observed that SLAMR could get into spines of different morphologies, although it becomes enriched at spines with PSD95 suggesting a preference for mature spines. Detailed analysis of transport suggested complexities in SLAMR transport and interaction with spines includes docking, undocking, and changing direction of transport at mature spines. What is particularly interesting is that often, SLAMR will change direction at spines which are already occupied by another SLAMR. This ability is diminished when critical protein binding regions, particularly the CaMKII binding region, of SLAMR are removed. This suggests that the neuron detects and regulates the abundance of SLAMR at spines and that protein-binding regions are important for this regulation. While, the physiological implications of these transport characteristics are less clear, our observations demonstrate that lncRNA could reside in spines and its localization undergo dynamic changes. Having a transport assay established, we next assessed whether KIF5C might transport SLAMR. We find that the loss of function of KIF5C results in a significant reduction in mobile SLAMR suggesting that KIF5C indeed mediates dendritic transport of SLAMR. Taken together, while the mechanism underlying motor protein-lncRNAs are unknown, these results suggest that different motor proteins are involved in mediating dendritic transport of lncRNAs.

The MS2:MCP system allowed us to examine how SLAMR behaves not just in basal conditions but also in stimulated conditions. We performed two-photon glutamate uncaging and observed MS2-tagged SLAMR dynamics in dendritic spines that exhibit structural plasticity and those that did not. Interestingly, spines that showed structural plasticity had an increase in the number of SLAMR moving toward the stimulated spine in the area surrounding the spine 5 minutes after stimulation. These results are consistent with the idea that SLAMR is necessary for structural plasticity and that stimulation increases the local abundance of SLAMR.

### SLAMR expression constrains CaMKIIα activity in synaptoneurosomes

A key step in obtaining mechanistic insights underlying function is the identification of interactors. Our unbiased analysis using pull-down assay combined with RNAseq and LC-MS/MS studies indeed revealed the complexity of SLAMR interactors. We find that these interactors including coding and noncoding RNAs and proteins presumably forming a multi-Protein-RNA complex. Among them, we identified CaMKIIα as a potential interactor. Considering the well-known functions of CaMKIIα in synaptic plasticity and memory (Lee et al., 2009), we next performed multiple experiments to validate CaMKIIα interaction and assess its significance. Independent experiments not only validated SLAMR-CamKIIα interaction, but also led to the identification of a 220nt fragment that is sufficient for interaction. Intriguingly, the deletion of this fragment showed that it is critical for dendritic transport of SLAMR. While this data is suggestive of the significance of CaMKIIα-SLAMR interaction for mediating SLAMR transport, considering the relative abundance (CaMKIIα is highly abundant compared to SLAMR expression levels), we considered the possibility that SLAMR might mediate the activity of CaMKIIα in dendrites. Consistent with this possibility, we find that loss of function of SLAMR results in a reduction in the active pool (T282 phosphorylated) of CaMKIIα in synaptoneurosomes. These results agree with the existing data that indicates how CaMKIIα is localized in dendrites and becomes translocated to spines in response to activity (Miller et al., 2002; Ortiz et al., 2017; Nihonmatsu et al., 2020). Taken together, these results suggest that SLAMR is a novel modulator of CaMKIIα function in hippocampal neurons.

### Memory specific role of SLAMR in dorsal hippocampal CA1

It is well known that the integrity of the dorsal hippocampus plays a crucial role in both associative and spatial learning and memory functions (Moser et al., 1993, 1995; Moser & Moser, 1998; Fanselow & Dong, 2010; Nakazawa et al., 2016; Victoria et al., 2016; Lee et al., 2019). However, SLAMR silencing in the dorsal CA1 did not interfere with spatial navigation learning and its long-term consolidation in MWM. This may mean that the specific role of SLAMR could be linked to precise forms of activation (Ex. Fear vs. Base-place navigation) on different subtypes of neurons in CA1 that have particular properties (Soltesz & Losonczy, 2018) and, in consequence, plays precise roles. This particular reasoning can be also applied to the CA3 hippocampal study (Soltesz & Losonczy, 2018). Previous studies have shown that while the CA1 is essential in both acquisition and consolidation of memory, the CA3 has been primarily related with fast acquisition responses (Lee & Kesner, 2004; Daumas et al., 2005; Vago et al., 2007; McHugh & Tonegawa, 2009). Supporting these observations, our results shown that during the early stages of acquisition SLAMR was enriched in CA1 but not in CA3, indicating that the increment of SLAMR is not necessary for CA3-related acquisition. Also, there were not significant changes in CFC memory consolidation when the expression of SLAMR was reduced in this particular hippocampal subarea.

Nonetheless, our FISH studies shown that SLAMR can be found in the entire hippocampal tri-synaptic circuit, especially in the soma of pyramidal neurons. Further, we found that this lncRNA is regulated by glutamatergic stimulation in primary hippocampal cell cultures supporting the *in vivo* results where the CFC training was able to induce the enrichment of SLAMR in CA1 dorsal neurons in the early stages of acquisition. It has been extensively demonstrated that CaMKIIα is crucial for excitatory synaptic transmission through NMDAR-dependent potentiation and, in consequence, determines learning and memory processes (Wang et al., 2017; Incontro et al., 2018). On the other hand, another SLAMR interacting protein Vimentin, an intermediate filament that stabilizes cytoskeletal interactions, is known to be involved in neurite outgrowth processes (Boyne et al., 1996; Wilhelmsson et al., 2019). The assembly of this protein is regulated by phosphorylation of several protein kinases at multiple sites. Specifically, several previous studies demonstrated that only CaMKIIα is implicated in the phosphorylation of Vimetin at its head domain Ser^82^. In addition, this phosphorylation is regulated in a calcium-dependent manner and seems to determine CaMKIIα translocation and its consequent function (Ogawara et al., 1995; Inagaki et al., 2000; Tsui et al., 2004, 2006; Ciani et al., 2011).

The memory specific role of SLAMR is intriguing. While SLAMR as well as its interactors are likely expressed in all cells, effect of its manipulation in dorsal CA1 produced a specific deficit in consolidation. This precision suggests a SLAMR specific cellular process in components of fear circuitry driving only for consolidation of contextual fear. As this is the first study to analyze in detail the role of a lncRNA in different types of memory and different memory phases at specific time-points, we do not have evidence of similar roles in other lncRNAs. Nonetheless, if we analyze previous findings from other laboratories using non-constitutive silencing with Antisense Oligonucleotides (ASOs) in specific brain areas, we find results that may indicate a similar pattern. For example, Gomafu and Adram are two lncRNAs well expressed in different cell populations in the brain and, specifically, in the PFC. However, the silencing of Adram before the fear conditioning training did not induce any differences in fear acquisition but impairs extinction (Wei et al., 2022). On the other side, silencing of Gomafu before the training, impairs fear acquisition while memory consolidation 24hrs after, is intact (Spadaro et al., 2015). These results suggest that lncRNAs may have specific roles in different groups of cells inside the same brain structure that are activated by inputs coming from diverse brain areas.

Together these results establish a new mechanism for activity dependent changes at the synapse and consolidation of contextual fear memory (Figure 9J). lncRNA SLAMR becomes transcriptionally upregulated in response to learning and hitchhikes onto molecular motor KIF5C for transport along microtubules to activated spine compartments for modulation of synaptic signaling and structural changes required for the consolidation. Loss of function or gain of function of SLAMR produce major impact on dendritic arborization, decreasing or increasing, respectively, the number of branches. Activity of SLAMR is mediated by complex interactions with other RNAs and proteins, especially, CAMKIIα. In summary, these results unravels the significance of lncRNA function at the synapse, and its spatial-temporal role in LTM .

## Materials and Methods

### Animals

For this study, we used C57BL6 adult male mice 8-10 weeks old provided by Jackson Laboratories. These mice were housed in groups of 5 and maintained on a 12hr light/dark cycle with *ad libitum* access to water and food. *In vivo* experiments were carried out during the light part of the cycle light/dark cycle. All *in vitro* experiments were performed in primary hippocampal cell cultures obtained from CD1 mouse pups, with the exception of MS2:MCP imaging experiments which were performed in primary hippocampal cell cultures obtained from sprague dawley rat pups. CD1 pregnant females were purchased from Charles River. Sprague Dawley pregnant females were purchased from Jackson Laboratories. Housing and experimental procedures were approved and supervised by the Institutional Animal Care and Use Committee of the Herbert Wertheim UF Scripps Institute for Biomedical Innovation & Technology and Max Planck Florida Institute for Neuroscience.

### RNAseq

#### Tissue preparation, LCM and RNA isolation

One hour after finishing the training in CFC or MWM, mice were sacrificed by a fast decapitation and the brains were removed, briefly washed in D-PBS, placed in cryomolds with OCT and frozen in dry ice and stored at −80°C (Kadakkuzha et al., 2015). Fresh frozen brains were coronal sectioned at 14μm on a Leica 3050s cryostat at −20°C after 1h of acclimation to this temperature. Dorsal hippocampal sections were mounted on PEN membrane slides (Leica), ~12 sections per slide with 3 slides per brain from a total of 6 brains. Staining and preparation of sections for the Laser Capture Microdissection (LCM) procedure was done using LCM staining kit (Life Technologies) with Cresyl Violet following manufacturer’s recommendations. Following staining and dehydration, slides were kept at room temperature for 15 min before LCM. The microdissection of the CA1 and CA3 hippocampal regions were done using the Leica LCM microscope (Leica LMD7000) where the laser power was set at 60 mW. Tissue was collected in RNAse free microtubes were 50μl of pre-chilled trizol were added just after finishing the micro sectioning. RNA was extracted using the trizol-Chloroform method and stored at −80°C before use.

#### Next generation sequencing and data analysis

RNA collected from the previous step was used for RNAseq. RNAseq analysis was carried out following the Wertheim UF Scripps Institute Genomics core protocol described in previous works from this laboratory (Kaddakuzha et al., 2015; Raveendra et al., 2018; Grinman et al., 2021). Analysis of the RNAseq results was performed using Tophat2 (v.2.0.9) and the Cufflinks suite (v.2.1.1). Trimming of the data was performed using the FastX-Toolkit (v.0.013) (Toolkit by Hannon Lab) (Raveendra et al., 2018). The alignment data were processed and quantified using HTSeq. The raw read counts generated in HTSeq were used to identify differentially expressed genes by using the Bioconductor DEseq for R. DEseq used the total size of each library to normalize the raw read counts and perform calculations on fold change and significance based on *p*-values and *p*-adjusted values. Comparisons were performed between samples of the three different CFC groups: Context+Shock, Context alone and Immediate shock mice. Transcripts were extracted from the Ensembl annotation file (NCBIM37).

### Interactome analysis

We used Metascape for pathway analysis (https://www.metascape.org) (Zhou et al., 2019) to analyze the list of significant different genes in common between Context vs. Context+Shock & Shock vs. Context+Shock conditions, all of them based on a pVal<0.05 and create an interactome analysis. A network with the enriched ontology clusters was visualized with Cytoscape. Each term was represented by a circle node proportional to the number of genes included in that term identified by a specific color.

### Quantitative real-time PCR (qRT-PCR)

The RNA from the LCM samples was reverse transcribed to cDNA using the same method previously reported from this laboratory (Kadakkuzha et al., 2013; Raveendra et al., 2018). 1 μg of RNA was used with Quanta cDNA SuperMix (Quanta Biosciences, Gaithersburg, MD) according to the manufacturer’s instructions and the expression of transcripts were quantified by qRT-PCR using SYBR Green PCR master mix (Applied Biosystems Carlsbad, CA) for detection in ABI 7900 cycler (Applied Biosystems Carlsbad, CA). Quantification of each transcript was normalized to the mouse 18S reference gene following the 2^−ΔΔCt^ method (Livak and Schmittgen, 2001; Kadakkuzha et al., 2013). One-way ANOVA and Student-Newman test was used to select genes with statistically significant expression levels.

### Localization studies of SLAMR

#### FISH

Mice were sacrificed by cervical dislocation and the brain was removed from the skull, briefly washed in pre-cold D-PBS, embedded in OCT and freezing in dry ice. Then, the fresh frozen brains were cryosectioned at 16μm on a Leica cryostat 3050 (Leica Systems). A DIG labeled ribo probe complementary to SLAMR lncRNA was prepared by in vitro transcription of cDNA templates by using SP6/T& RNA polymerases (DIG RNA labeling Kit, Sigma). Sense and Antisense strands of 250nt were prepared by PCR using mouse hippocampus cDNA as a template and transcript specific PCR primers and ligated to pCRII-TOPO Vector with dual promoters SP6 (for antisense strand) and T7 (sense strand).

After the sectioning the tissue was dried and acclimated at RT for 2 hours, washed with D-PBS fixed with a 4% PFA solution for 10 min at RT, washed again with D-PBS (3 times, 3 min each), wash with 0.2% glycine in D-PBS (5 min), D-PBS washes (2 x 5 min), acetylated in TEA solution for 10 min, pre-hybridized at 68°C for 1 hr and hybridize with the probes overnight (approx. 16 hrs). After hybridization the signal was visualized using the TSA Plus Fluorescein Systems from PerkinElmer (TSA plus Cyanine detection kit, Akoya) for DIG detection. Images were acquired by using Zeiss LSM 880 with Airyscan confocal microscope system.

### Fractionation

Fractionation experiments were carried out using C57BL6 adult mice hippocampus lysates. The SurePrep^™^ Nuclear or Cytoplasmic RNA purification kit (Fisher Scientific) was used following the manufacture’s manual. The RNA isolated was reverse transcribed and RT-qPCR were performed following the procedures described before in these methods. Actin mRNA that is enriched in the cytoplasm was used as a control of the fractionation as well as normalization element. Following the same procedure previously published from our lab. CT values of each lncRNA were normalized to Actin, resulting in a ΔCT value. Relative differences between lncRNAs and Actin were determined by subtracting the ΔCTs of nucleus fraction from the ΔCTs of cytoplasm fraction for each sample, resulting in ΔΔCT values (Kadakkuzha et al., 2015).

### Primary Hippocampal Cell Cultures

Neuronal cultures from hippocampus were obtained from brains of 21-day mice embryos following the same procedure described before from our laboratory (Raveendra et al., 2018). Embryonic brains were removed from the skulls and membranes cleaned. Then, brains were dissociated using papain (29.5 U/mg protein, Worthington). The cells obtained were plated in poly-D-lysine (PDL) treated plates with a 5x10^5^ density for experiments that required RNA isolation or a density of 1x10^5^ in glass coverslips for imaging experiments. Both types of cultures were maintained in Neurobasal medium (Invitrogen), penicillin/streptomycin and 2% B27 (Invitrogen) at 37°C in 5% CO2.

### Loss of function analysis using Gapmers

Three different antisense LNA Gapmers against 2610035D17Rik and a Negative Control Gapmer were design by EXIQON (QIAGEN) and synthesized for *in vitro* (Gapmer_1: GGACAGGTCAATGGCG; Gapmer_2: TGATGTGAGTTTCTAC, Gapmer_3: ACAAACGAGATAGTAG) and/or *in vivo* experiments. (Gapmer_1: GGACAGGTCAATGGCG *in vivo* purified, named as: SLAMR_Gapmer in this work)

#### In vitro efficiency:

Gapmers efficiency were first tested in primary hippocampal cell cultures. Gapmers were transfected to primary hippocampal neurons (7-14 days in vitro (DIV)) using Lipofectamine RNAiMAX according to manufacturer’s guidelines. For the transfection 6 pmol of Gapmer was mixed with 1μl of Lipofectamine RNAi Max in 100μl of Neurobasal medium and incubated for 15 min. The complexes of RNAi and Lipofectamine RNAiMAX were added to the cells in incubation at 37°C. 48 hrs and 72 hrs after the transfection with each Gapmer, cells were washed with PBS and collected into trizol to proceed with the RNA extraction using the trizol-chloroform protocol. Reverse transcription and RT-qPCR was performed following the same method described before in this work.

#### In vivo efficiency:

We decided to determine the efficiency *in vivo* of Gapmer_1 (SLAMR_Gapmer for behavior) before the behavioral performance. For this, 8 weeks old C57BL6 mice were bilaterally infused in dorsal-Hippocampus CA1 area with a single infusion of *in vivo* ready SLAMR_Gapmer or a Negative control using the transfection reagent JetSI (Polyplus-transfection S.A, Illkirch, France) (25pmols, 0.5μl per side at 0.1μl/min delivery rate). Using the following coordinates: AP=−2, DV=−1.5, L=±1.5. 72hr after the infusion mice were sacrificed and the CA1 dorsal-hippocampal area was dissected and fast freezing in dry-ice. The tissue collected was processed using the kit RNA Aqueous 4PCR kit (Ambion, Invitrogen). 200μl of lysis buffer form this kit was used to grind the tissue and we follow proceed according to the manufacturer’s instructions. The RNA obtained was reverse transcribed and the levels of SLAMR measured by RT-qPCR following the same method described before.

### Constructs & Transfections

For lipofectamine transfection, about 1.50 x 10^5^ cells/well primary mouse hippocampal neurons were plated on PDL-coated CellVis glass-bottom 24-well plate (P24-1.5H-N). For pLL3.7-GFP scramble control (Addgene #11795), SLAMR-shRNA (#CS-GS1731L, Genecopeia), TET-SLAMR (#CS-SH1343L, Genecopoeia), and OE-SLAMR (#CS-GS1731L-Lv235-01) arborization analysis were transfected 68-74 hrs prior to the imaging (DIV 14) using Lipofectamine 2000 (Thermo) following manufacturer’s instruction. Before adding the DNA-lipofectamine mixture, half of the conditioned culture medium was removed and saved for later. Four hours after incubation with DNA-lipofectamine mixture, the medium was removed and immediately added 500ul of 1:1 of conditioned/fresh medium. Right before imaging, all culture medium was replaced by 500 ul of Hibernate E low fluorescence buffer (BrainBits) to maintain the ambient pH environment.

For magnetofection, about 6x10^4^ cells/dish primary Rat hippocampal neurons were plated on the center of a PDL-coated coverglass region of MatTek dish (P35G-1.5-14-C). For SLAMR-MS2: MCP (Plasmids: MS2-SLAMR #CS-CC1532L, Genecopoeia; tdMCP #98916 addgene; RFP-MCP #64541 addgene; MS2-SLAMRΔ92-289, CS-CC1532L-02 Genecopoeia; MS2-SLAMR-Δ898-1130 #CS-CC1532L-03, Genecopoeia) imaging, hippocampal neurons were transfected 12-16 hrs (DIV14-22) prior to imaging using Combimag (OZ biosciences) and Lipofectamine 2000 (Invitrogen) according to the manufacturer’s instructions. Briefly, 1.2 μg DNA was incubated with 0.5 μl lipofectamine in 50 μl transfection medium (Neurobasal media supplemented with 2 mM Glutamax without B27 or antibiotics) for 5 min, then mixed with 0.5 μl Combimag diluted in another 50 μl transfection medium for 10 min. The DNA-lipofectamine-Combimag mixture was further diluted in 125 μl of transfection medium. Then, the conditioned medium from cultured neurons was harvested and the neurons were immediately rinsed twice in warm transfection medium. Transfection medium used for rinsing was removed and 150 μl of DNA-lipofectamine-Combimag mixture was added to the neurons. Neurons were placed on a magnetic plate for 20 min inside a 37°C and 5% CO2 incubator. After 20 min, neurons were rinsed once in warm transfection medium and replaced with the previously harvested warm, conditioned medium. Right before imaging, all culture medium was replaced by 1 ml of Hibernate E low fluorescence buffer (BrainBits) to maintain the ambient pH environment.

### Lentiviral production

pLL3.7-GFP-ShLenti against SLAMR (SH1343L) and pLL3.7-GFP scramble control (Addgene #11795). Each plasmid was co-transfected with REV, MDL packing vectors and vesicular stomatitis virus (VSVG) envelop vector from Addgene into HEK293T cells using TransIT transfection reagent (Mirus) for lentiviral particles production. Supernatant was collected at two different time points 36 and 72 hrs after transfection. The lentiviral particles were concentrated and pelleted through sucrose cushion and re-suspended in sterile PBS and storage at −80°C. Viral titer was determined by measuring GFP fluorescence after infection of HEK293T cells with serial dilutions of the virus, suing flow-cytometry (BD biosciences C6 Accuri and InterlliCyt sampler powered by FlowCyt software). Our lab-made SLAMR-OE lentivirus had a titer of 3.9x10^9^ IFU/ml. Vigene synthesized NC-GFP and SLAMR-KD lentiviruses at titers of 1.07x10^9^ IFU/ml and 9.75x10^8^ IFU/mL respectively.

### SLAMR-MS2: MCP transport timelapse video microscopy

Live imaging of neuronal cultures was carried out using a state-of-the-art inverted Spinning Disk Confocal microscope (3i imaging systems, Yokogawa CSU-W1 confocal scanner unit) with 4 laser lines (405 nm 20 mW; 488 nm 50 mW, 561 nm 75 mW and 638 nm 75 mW) connected to a charged coupled device confocal camera (Andor iXon Life 888). The temperature of the sample stage was maintained at 37 °C by a Okolab Boldline Stage Top Incubation system. The image acquisition was controlled by SlideBook6 software. For MS2-SLAMR: MCP-tdGFP transport recording and PSD95-mcherry imaging, neurons were co-transfected and imaged within 12-20 hrs in Tyrodes buffer (in mM: 119 NaCl, 2.5 KCl, 2 CaCl2, 2 MgCl2, 25 HEPES, 30 d-glucose; pH 7.4). Time-lapse confocal imaging of dendrites at least 10μm away from the soma was performed with a 63x oil objective (1.46 N.A.). For each neuron, a movie was first captured in the 488 Channel at 600 ms exposure and at a rate of 1-1.03 frames/s for a total recording time of 300 s. Promptly after finishing this recording, a movie would then be recorded in the 561 channels at 100 ms exposure at a rate of 10 frames/s for a total recording time of 120 s.

Time-series image data of reporter mRNAs was analyzed by kymographs. Dendritic 100-μm segments at ≥20 μm from the cell body were selected. The KymoResliceWide plugin of ImageJ was used to generate kymographs and to trace tracks. Velocities were calculated from the angle of the traced tracks where Velocity (μm/s) = TAN (π/180 × θ) × fps/(pixels/μm); fps = frames per second. Only movements greater than 1.5 μm were considered for analysis. Tracks were terminated when a particle stopped, changed direction or left the region of interest (ROI). Average speed and displacement were obtained by calculating the mean. Anterograde and retrograde tracks were counted to calculate the percent of anterograde transport. The sum of anterograde and retrograde displacement lengths was used to calculate the percent of total anterograde displacement. Dual-color kymographs were generated by overlaying identical regions of interest from two separate channels. Events in dual-color kymographs were manually selected and distances were manually measured in ImageJ (line tool). For the MS2-SLAMR : spine interaction studies, MS2-SLAMR and dendritic spines were considered to have an interaction with each other if the MS2-SLAMR (green track) came within 1 μm of a PSD95 puncta (red track) based on the kymograph. Data were processed and subjected to statistical analyses in GraphPad Prism 9.

### MS2-SLAMR Two-photon glutamate uncaging experiments

The neurons were transfected with MS2-SLAMR: MCP-tdGFP and RCaMP1.07 16-20 h prior to imaging. RCaMP1.07 fluorescence was used to identify responsive spines for glutamate uncaging. Prior to single spine stimulation, the imaging media was replaced with a modified Tyrodes buffer (as above but with 4 mM CaCl_2_ and no Mg^2+^) containing 1 uM TTX (citrate salt, made in water) and 2 mM 4-Methoxy-7-nitroindolinyl-caged-L-glutamate (MNI caged glutamate; Tocris Bioscience, 100 mM stock made in the modified Tyrodes buffer). Glutamate uncaging was performed using a multiphoton laser at 720 nm (MaiTai HP 1040S) and a pockles cell (Conoptics) for controlling the pulses. Simultaneous confocal imaging and two-photon glutamate uncaging was performed using the SlideBook6 software in association with the MaiTai 2x software for controlling the laser beam. Spines (primarily mushroom-shaped) positioned at least 50 μm away from the soma, on secondary or tertiary branches, were selected for these experiments. Baseline time-lapse recordings of the GFP (600 ms exposure) and RFP (100 ms exposure) fluorescence were acquired for 1 min at a rate of 1.2 frames/s prior to glutamate uncaging. To test if a spine responds to uncaging, an uncaging spot (~ 1 x 1 pixel or ~ 1 um^2^) was placed near the spine head and one to two stimulation pulses were given at a pixel duration of 10 ms and with a laser power of 1 mW. The spines showing specific localized calcium transients measured by an increase in RCaMP1.07 fluorescence was chosen for the experiment. An uncaging protocol of 30 pulses (one pulse every alternate second, total 60s) at 0.5 Hz with 10 ms pixel duration and 1 mW laser power was used. Immediately following uncaging, another 45s of continuous time-lapse frames were recorded at the same frame rate as above. For the analysis of change in RNA granules following stimulation: Within 60s of completing stimulation, time-lapse movies with 3D Z-stacks (11 slices, ~ 5 um depth and 0.5 um step size) were acquired in both the GFP (600ms) and RFP (100 ms) channels. The same Z stack time-lapse (11 slices, ~ 5 um depth and 0.5 um step size) imaging was repeated every 1 min until 5 min after stimulation, then every 2 mins until 30 min, and continued every 5 min until 60 min after stimulation. For the analysis of SLAMR velocity and trajectory: a 2min time-lapse movie at ~1.1 fps was taken in the green channel (600ms) before stimulation, then stimulation occurred as above while taking time-lapse movie in the red (100ms) and green (100ms) channel, followed immediately by a single frame in the red channel and a 5min time-lapse movie at ~1.1 fps was taken in the green channel (600ms), then every ten minutes a 2min time-lapse movie at ~1.1 fps was taken in the green channel (600ms) until 50min was reached.

### Synaptic protein extraction

Primary cultured hippocampal neurons on 6-well plates were processed for synaptoneurosome preparation after transfections. Neuronal culture medium was carefully removed, and after two rinses in ice-cold PBS, cells were lysed manually in Syn-Per buffer (Syn-PERTM Synaptic Protein Extraction Reagent) supplemented with 1 protease inhibitor cocktail tablet and 100 μL of phosphatase inhibitor cocktail 1 and 2 each. Samples were centrifuged at 1200 xg for 10 min at 4°C, the pellet was discarded, and the supernatant was transferred to a new tube. Thirty microliters from the sample of the supernatant were saved as homogenate for analysis. The supernatant was centrifuged at 15,000 xg for 20 min at 4°C. The supernatant was removed from the synaptosome pellet and saved as the cytosolic fraction for analysis. The synaptosome pellet was suspended in 30 μL Syn-PER.

### Morphology Assessments

After 72 hrs of transfection and 24hrs of incubation with 0.5ug of Doxycycline, hippocampal neurons using shRNA plasmid expressing NC-GFP, TET-SLAMR, and OE-SLAMR images of dendrites were collected at 36°C in the light microscopy facility at UF Scripps Biomedical Research, using a confocal microscope (FV1000; Olympus; Apo N 60X/1.49 Oil) in Hibernate-E (Brainbits). Z-stack images were acquired using Fluoview1000 (64 bit) software (Olympus) and converted into a maximum projection intensity image in FIJI (ImageJ, NIH). Dendritic arbor was quantified via the Sholl analysis plugin in FIJI. The center of soma is considered as the midpoint and origin of the concentric radii was set from that point to the longest axis of soma. The parameters set for analysis were: starting radius 20 μm, ending radius 100 μm, radius step size 10 μm. The maximum value of sampled intersections reflecting the highest number of processes/branches in the arbor was calculated and the number of intersections plotted against distance from the soma center in μm. Data was analyzed using Two-way ANOVA. Spine morphology was analyzed using MATLAB software developed in the light microscopy facility at the Max Planck Florida Institute. By using a geometric approach, this software automatically detects and quantifies the structure of dendritic spines from the selected secondary branch (100 μm length) in the Z-stack confocal image. The software assigns the detected spines to one of the three morphological categories (thin, stubby or mushroom) based on the difference in structural components of the spines i.e., head, neck and shaft. Student’s t test was carried out to evaluate the statistical difference amongst the groups.

### Two-photon fluorescence microscopy and two-photon glutamate uncaging for analysis of spine morphology

MNI-caged glutamate uncaging and timelapse structural imaging of spines were performed using a custom-built two-photon (2p) laser microscope as previously described (Colgan et al., 2018). 2p-imaging and uncaging was performed using two Ti-sapphire lasers (Coherent, Cameleon) at wavelengths of 920 nm (1.45–1.55 mW under the objective) for imaging and 720 nm (3.0–3.5 mW under the objective) for uncaging. Green fluorescence emission was collected using an immersion objective (LUMPlan FL N 60×, numerical aperture 1.0, Olympus), reflected by a dichroic mirror (565 nm LP) and passed a filter (Chroma, 510nm/70-2p) before entering the fast photoelectron multiplier tubes (PMT) (H7422-40p; Hamamatsu). Fluorescence images were acquired and quantified using TimeHarp 260 Pico card (PicoQuant, Inc) and custom-built software, FLIMage (Ver 2.0.20) written with #C (https://github.com/ryoheiyasuda/FLIMage_public). Fast-rate simultaneous image acquisitions with uncaging were collected by 128 × 128 pixels at a single z plane without averaging per frame (frame rate 3.91 Hz). Image acquisitions for slow-rate imaging were collected by 128 × 128 pixels as a z stack of five frames with 1 μm distance in each frame and averaging 6 scans per frame (frame rate 0.65 Hz). Maximum intensity projection images were generated by a z-stack of five frames for slow-rate imaging. MNI-caged L-glutamate (4-methoxy-7-nitroindolinyl-caged L-glutamate, Tocris) was uncaged with a train of 8–10 ms laser pulses (under the objective, 30 times at 0.5 Hz) near a spine of interest. Experiments were performed at room temperature (24–26 °C) in ACSF solution containing: NaCl (127 mM), KCl (2.5 mM), NaHCO3 (25 mM), NaH2PO4 (1.25 mM), CaCl2 (4 mM), glucose (25 mM), tetrodotoxin (1 μM), and 4-MNI-caged L-glutamate (4 mM), bubbled with 95% O2 and 5% CO2. We examined secondary or tertiary branches of apical dendrites of cultured hippocampal neurons at 21–25 days in vitro. Spine volume change was calculated by F/F0, in which F0 is the average spine intensity before stimulation. All values are presented as mean ± SEM. Number of independent measurements (n=[spines/neurons]). Mann-Whitney’s U test and Two-way ANOVA, followed by Turkey’s test were used to compare grouped data sets for fast and slow rate imaging, respectively (Prism 9.4.1, GraphPad). Data were excluded if signs of poor cellular health or procedural artifacts were apparent (for example, dendritic blebbing, and displacement of dendrites).

### Puromycin Labeling

DIV14 hippocampal neurons were either transfected with SLAMR-OE or control NC-GFP via lipofection. After 72 hours, prior to ICC, neurons were treated with Puromycin for 15 min to block protein synthesis and label newly synthesized proteins. Then they were fixed with 4%PFA in PBS, washes 3 times and blocked with 10% horse serum in PBS-T (1%) for 1 hour. After blocking, neurons were labelled with anti-GFP antibody (chicken), anti-Puro antibody (mouse), anti-Tub antibody (rabbit), and incubated overnight at 4°C. After 3 washes, the cells were incubated with the corresponding secondary antibodies tagged with Alexa 488, Alexa 561 and Alexa-647. After 3 more washes, the slides were mounted, with a mounting medium containing DAPI to identify cells. Images were captured in Olympus FV 3000 with an average of 15 stacks per image in 3 channels (green, red and far red).

After imaging, the Intensity calculator plugin was used in ImageJ to quantify the Corrected Total Cell Fluorescence (CTCF) from both the cell body and randomly selected areas of dendrites of neurons.

### RNA pull-down assay: Proteomic, transcriptomic and protected fragments analysis

Sense SLAMR RNA sequence was full-length cloned for this experiment into a pCR-II-TOPO vector from a template (Thermo Fisher), linearized and 5’ end Biotin-labeled (Roche) by *in vitro* transcription using a SP6 RNA polymerase enzyme for the sense strand and T7 for antisense strand that was used as a control.

C57BL6 mice brains (8 weeks old, n=6) were used for this study. The tissue was processed for pull-down following the same procedure described before. However, the samples were not incubated with RNAse A. After the streptavidin beads incubation, these were washed with high stringency lysis buffer, diluted in trizol and incubated for 40 min in rotation. Them, the RNA from the samples were isolated using the Direct-zol RNA miniprep kit (Zymo) according to the manufacture’s manual. The RNA was processed for RNAseq and analyzed following the same procedure described before by the TRSI Genomics Core.

#### Protected fragments

Protected fragments study was carry-out following the pull-down protocol described for the RNA-protein interaction. After RNAse A incubation the samples were diluted in PBS and the protected fragments purify and concentrated using sucrose cushion. Then, the supernatant was removed, and the RNA fragments pellet diluted in Trizol reagent for their purification using the trizol-chloroform RNA purification method. The samples were sequenced by the Scripps Genomics core using HTseq and the reads alignment to transcription using Salmon software.

#### Proteomics

For proteomics studies, the hippocampus dissected and grinded in lysis buffer (Tris-HCl 50mM, NaCl 150mM, EDTA, 0.5M, NP-40 0.25%, DTT 1mM, Protease inhibitor (Sigma), Phosphatase inhibitors cocktails 2 & 3 (Sigma), Superase-In RNAse inhibitor (Thermo fisher), UltraPure BSA (MCLAB). Tissue was incubated in a rotator with the lysis buffer for 1 hr to finish the lysate and centrifuge at 1200xg for 20 min to remove the debris. Hippocampal lysate supernatant was transferred to a new tube and incubated with 2μl of the sense or antisense biotinylated probes for 2 hrs. During that time, the streptavidin-coated magnetic beads (NEB) were washed with the same lysis buffer and blocked with yeast tRNA and glycogen (0.2mg/ml) for 1 hr. Blocking medium was replaced for lysis buffer before use. 10ul of pre-cleared magnetic beads were added to each sample and incubated in slow rotation for 1 hr. After finishing the incubation, the beads were washed 3 times with the lysis buffer and plated in a 24 well plate for UV/Cross-link (320 mJ/cm^2^). Then, the samples were placed again into 1.5 ml tubes and washed with a high stringency buffer (Tris-HCl 50mM, NaCl 600mM, EDTA 0.5M, NP-40 0.25%, DTT 1mM, Superase-In RNAse inhibitor, Protease inhibitor, Phosphatase inhibitor cocktails 2 &3). Finally, the beads with the RNA/protein complexes were diluted in Tris buffer + 0.15% SDS + 1 ul RNAse A (10ug/ul, Thermo Fisher) and incubate at 37°C for 1 hr. Bound proteins were eluted by heating to 96°C in the presence Laemmli sample buffer for 10 min for denaturalization.

After finishing this process, the samples were loaded in a pre-cast Tris-glycine polyacrylamide gradient gel (4-15%, 1mm, Bio-rad) to perform a denaturing SDS-PAGE. The gel was washed with HPLC water, fixed with a solution of 50% Ethanol-5% Acetic Acid for 1 h. After that the bands were sectioned for each sample (sense and antisense). The gel was then washed, reduced, alkylated, tryptic digested and ZipTip cleaned for LC-MS/MS by TRSI Proteomics Core.

#### Western-Blot/Immuno-Blot

For the WB analysis, the protein concentration was determined using a BCA kit. Protein (10-25 μg) was used for WB analysis. The antibodies used are listed in the key resources table. The target proteins were detected using anti-rabbit or anti-mouse secondary antibodies at a 1:5000 dilution and then visualized by chemiluminescence (Amersham Biosciences, Piscataway, NJ). The autoradiograms were analysed by ImageJ.

### Immunocytochemistry (ICC)

ICC experiments were performed using primary hippocampal cell cultures in coverslips on DIV14 and/or 72hrs after the transfection. Briefly, culture media was first removed to wash the cells with PBS and fixed in a solution of PBS + 4% PFA (pH 7.4) for 10 min, then wash again 3 times with PBS. After complete removal of PFA, neurons were permeabilized in 0.5% TritonX-100 diluted in PBS for 10 min and incubated in 5% normal goat serum (NGS, Sigma) in PBS for 1 hr and incubated with primary antibody (see key resources) at 4°C over-night. The day after cells were rinsed 3 times in PBS to remove the primary antibody and incubated with the appropriated secondary Alexa antibodies 594, 488 or 405 (1:500 Molecular Probes) 1h at RT. Finally, the cells were washed with PBS and mounted in slides using Fluoro gel II (Electron Microscopy Sciences) for imaging.

### Mouse Behavior Assays

Before starting each behavioral training, mice were acclimated to the transportation and waiting room for one hour followed by a handling manipulation of 1 min per mice for three days.

#### Basal behavior

To analyze transcriptional changes in different hippocampal subregions induced by learning/memory processes, two different experiments were carry-out:

##### Contextual Fear Conditioning (CFC):

CFC was performed using a modified version of Noldus PhenoTyper Model 300 chambers (30x30x40) (Leesburg, VA) that includes a grid floor (30x30) prepared with a shock delivered system (Shock Scrambler ENV-414S; Med Associates, ST. Alvans, VT) and equipped with a white light inside (Rizzo et al., 2017). Automated tracking and shock delivery control were performed using EthoVision 8.5 software (Noldus Information Technology, Leesburg, VA). Each chamber was cleaned with 70% ethanol before and after finishing each training session. 72db white noise was played in the room to mask any unintended noise that may be add to the context. During fear conditioning training session mice received three 2 s 0.75 mA scramble foot shocks 2.5, 3.5 and 4.5 min after placement into the chamber. Mice were promptly removed from the chamber after 5.5 min. Mice were divided into 3 different groups: those who received the training with the three shocks (Context + Shock group), those who were exposed to the context without the shock (Context alone group) and one-third group received a single and immediate shock (2s) and were fast removed from the box (Immediate Shock group) (Rizzo et al., 2017). All the mice were sacrificed 1 hr after finishing their respective training.

##### Morris Water Maze (MWM):

MWM consisted of a circular tank of 120 cm diameter filled with 22°C water located in a white light-illuminated room with visible external cues. Water was colored with a non-toxic white paint to hide the position of the platform. The escape platform consists in a 5 cm diameter plastic circle that was submerged 1.5 cm under the water and separated 8 cm from the tank wall. During acquisition/training trials mice were trained to escape from water by swimming from variable starting points around the tank to the hidden platform and allowed to remain there for 15s with a limited exploration time of 1 min for each trial of a 4 total training trials a day. After each trial, mice were dried and returned to their home cages. All sessions were recorded by a video camera located above the tank and analyzed using the tracking software EthoVision 8.5. For basal line experiments mice were divided into three different groups: 1 week of swimming training, 1 day of training and swimming session without platform. All the mice were sacrificed 1 hr after finishing the training.

#### Genetic manipulation of SLAMR

8 weeks old C57BL6 mice were stereotaxically implanted with bilateral cannulas for CA1 or individual cannulas for CA3. Mice were anesthetized with 1% Isoflurane and secured in a Kopf stereotaxic apparatus. For dorsal CA1 infusion, bilateral stainless-steel guide cannula (26G, Plastics one) were implanted using the coordinates AP= −2, ML=±1 and DV =−1 (from the surface of the skull on bregma). For dorsal CA3 infusion, individual stainless-still guided mini cannulas (26G, Plastics one) were implanted on each side of the brain using the coordinates AP=−1.95, ML=±2.5 and DV=−1.5. Clearance through the guide cannula was maintained with 33G obturators (Plastics One) without projection beyond the tip of the cannula and protected with a dust cap (Plastics One). After surgery, animals were given 1 week of recovery during which they were monitored daily to check their health condition. Animals with abnormal motor behavior were excluded from the experiment. Gapmer infusions were delivered into the target area using an infusor with 1 mm of projection below the cannula, 0.5ul of Gapmer (25 pmol per side, 50 pmol total) was delivered in a 0.1ul/min ratio and let 5min for diffusion per side of the mouse hippocampus. 3 days before the infusions mice were acclimated to the procedure room for 1 hour following by 1 min of handling and the manipulation of the cannula (remove/replace caps and dummies) to habituate the mice to this manipulation.

##### CFC:

Three different experiments were designed to study the role of D17Rik in different memory aspects: Acquisition, Consolidation, Extinction and Recall. Each of these experiments count with 3 different experimental groups: Sham, Negative Control Gapmer and SLAMR_Gapmer. Training was performed following the same protocol described before in this work. For *acquisition* evaluation, mice were tested in the same context 1 hr after finishing the training without any shock delivery. Learning was measured by total percentage of freezing in blocks of 1 min for 5 min total. For long-term memory *consolidation*, mice were test in the same context 24 hrs after training without any shock delivery, measured by total % of freezing behavior for 5 min in blocks of 1 min. For *extinction*, mice spent 30 min in the same context of training without any shock delivery. The extinction was measured by % of freezing time by blocks of 5 min for 30 min total. *Recall test after extinction* was measured 24hr after finishing the extinction training by measuring the freezing time during a 5 min test in blocks of 1 min.

##### MWM:

For this experiment mice were divided into three different groups: Sham, Negative Control Gapmer and SLAMR_Gapmer. 72hrs after the Gapmers infusion, All the mice receive 7 days of training with 4 trials each day. 24hrs after finishing the training the long-term memory consolidation was evaluated in a single test session. For this test, the platform was removed from its position and the mice were placed in a new started point for a single trial of 1 min. The total latency time, distance and velocity was used as a measure of leaning and the time spent in the target quadrant of the platform was used to indicate the long-term memory consolidation.

#### Histological analysis

To verify the position of the cannula and the point of delivery location for the reagents, we perform a cresyl violet staining and light microscope analysis. After finishing each experiment all the mice were sacrificed and the brain removed from the skull and placed on 4% PFA solution. 24hr after, the brains were transferred to a 30% sucrose solution for 48hrs and stored in cryomolds with OCT compound (Tissue-Tek, Sakura Finetek) at −80°C before sectioning. Then, the hippocampus was cryosectioned in 35μm coronal slides and mounted in superfrost pre-coated slides (Fisher brand). To localize the placement of the cannula and the infuser the slides were stained following the cresyl-violet method and analyzed by light-microscope (AF6000 Leica Microsystem). Only those mice who present a bilateral infusion in the dorsal CA1 were included in the study.

### Statistical Analysis

Levels of significant in this study are based on p-values calculated by GraphPad Prism 8 or 9 (Graph Pad Software) and derived using Student’s t test, Mann-Whitney U test, One-way or Two-way ANOVA. Tukey, Dunnett, or Sidak tests were used for post hoc analyses. Significance was defined as p<0.05. The details for each experiment as well as the number of replicates and statistical specifications are indicated in the figure legends, results, and supplementary tables.

## Supplementary Material

1

## Data Availability

RNAseq data related to Figures 1 and 6 were deposited to NCBI Gene Expression Omnibus with the accession numbers GSE214838 and GSE214839 respectively, and all relevant data are available from the authors upon reasonable request.
