# Supplementary material for: SLAMR, a synaptically targeted lncRNA, facilitates the consolidation of contextual fear memory
Source: Res Sq. 2023 Mar 14:rs.3.rs-2489387. Preprint. [Version 1] doi: 10.21203/rs.3.rs-2489387/v1 (PMC10055528; doi:10.21203/rs.3.rs-2489387/v1)
Supplement: 1 [file NIHPPrs2489387v1-supplement-1.pdf]

SUPPLEMENTARY INFORMATION

SLAMR, a synaptically targeted lncRNA, facilitates the consolidation of contextual fear memory

Isabel Espadas[1#], Jenna Wingfield[1#], Eddie Grinman[1], Ilika Ghosh[2], Kaushik Chanda[1], Yoshihisa Nakahata[2], Karl Bauer[3], Bindu Raveendra[1], Michael Kiebler[3], Ryohei Yasuda[2], Vidhya Rangaraju[2], Sathyanarayanan Puthanveettil[1*]

8 Supplementary Figures (S1-9)
9 Supplementary Tables (S1-11)
24 Supplementary Movies (S1-24)

SUPPLEMENTARY FIGURES

Supplementary Figure S1. D17Rik KD does not affect the expression of its neighbor genes A. Experimental design for training for the C+S and Context alone groups. Fear learning is represented by the percent of freezing time after each shock. **B-D.** GO analysis of cellular component (B), biological process (C) and molecular function (D). These results are based on the analysis of significant genes in the RNAseq data from C+S condition that overlap between C+S vs Context and C+S vs Shock comparisons. **E.** Chromosome 11 with D17Rik lncRNA enlarged to show its location and its structure. **F.** UCSC genome browser with PhyloCSF 6-frame translation indicate no predicted open reading frames for D17Rik. **G.** CPAT analysis. Coding Potential Assessment tool identified a single ORF with non-coding potential in D17Rik. (**H-J**) Pictures show genomic regions adapted from NCBI for the lncRNAs: **H.** D17Rik in mouse chromosome 11. **I.** LncRNA 00673 in human chromosome 17, and **J.** LncRNA SlincR in Zebra Fish chromosome 3. **K**. Schematic representation of D17Rik locus in mouse chromosome 11. **L.** Heat-map representing significant changes in the expression of genes in D17Rik locus detected in CA1 after CFC by RNAseq. **M**. Plot indicating long2Fold changes in D17Rik and Sox9 between different groups after fear conditioning. **N.** *In vitro* study of genes in the D17Rik locus study after KD D17Rik in hippocampal cell cultures (n=3 per condition, per group). **O.** *In vivo* study of genes in D17Rik locus after silence D17Rik in dorsal CA1 of hippocampus (*$p<0.05$, unpaired student t test, n=7). **P**. Fluorescence in situ hybridization (FISH) show that the lncRNA D17Rik is expressed in pyramidal layer of CA2 and CA3 and hilus of mouse hippocampus. High magnification details from pyramidal layer in dorsal CA1 indicate a mainly cytoplasmic subcellular localization of D17Rik. Confocal microscopy photomicrographs show D17Rik signal in red and DAPI signal from the nucleus in blue. Scale bars = 100 µm. **Q.** These observations are also supported by fractionation studies that show a significant enrichment in the cytoplasm of D17Rik in these neurons compared to the nucleus using Actin levels as a reference-normalization element (*p-value <0.05, **p-value= 0.0005, Student t Test, n= 3).

Supplementary Figure S2. A. FISH showing colocalization of SLAMR and GFP in a distal dendrite of a primary hippocampal neuron transfected with MS2-SLAMR and MCP-GFP. White arrowheads point to regions of colocalization. **B-C.** Cell body images (top), dendrite(left) and kymographs(right) of neurons transfected with MCP alone or MS2-SLAMR+MCP. Red arrows point to the base of the selected dendrite. **D-E.** Cell body images (top), dendrite (left) and kymographs (right) of neurons transfected with MS2-SLAMR+MCP(green) and PSD95-mCherry(red). Red arrows point to base of the selected dendrite.

Supplementary Figure S3. A-D. Replicative cell body images, dendrite, and kymographs of neurons transfected with MS2-SLAMR+MCP-RFP(red) and control scrambled Scr-shRNA (green) (**A-B**) or KIF5C-shRNA(green) (**C-D**). Green arrowheads point to anterograde tracks, red arrowheads point to retrograde tracks. White arrows in cell body image point to base of the selected dendrite. **E.** qPCR analysis validating SLAMR knockdown in cultures transduced by shSLAMR virus. Error bars=SEM. One-way

ANOVA+Dunnett's test, **p<0.01. **F.** qPCR analysis validating SLAMR enrichment in cultures transduced by SLAMR-OE virus. Error bars=SEM. One-way ANOVA+Dunnett's test, ****p<0.0001. **G.** Quantification of spine width during the time-course of the 30 pulse glutamate uncaging experiment, normalized to the average of the –1.25 and 0 min timepoints. Error bars=SEM. Two-way ANOVA+Sidak's multiple comparisons test. *p<0.05, ***p<0.001, ****p<0.0001. **H-I.** Representative dendritic region and kymographs during after the stimulation experiment of an unresponsive (**H**) and responsive spine (**I**). Red arrowheads in the dendrite point to the stimulated spine, green arrows point to the tracks moving toward the stimulated spine.

Supplementary Figure S4. Validation of Synaptoneurosome Isolation. A. Western blot analysis of total homogenate from primary hippocampal neurons, the cytosolic fraction and the synatoneurosome fraction, probed for GluR2, Synaptophysin, and β-actin. **B, C.** Quantification of A, **normalized with β-actin.** One-way ANOVA+ Tukey's multiple comparison. *p-value<0.01, **p-value<0.05, ***p-value<0.005. Error bars=SEM.

Supplementary Figure S5. Pull-down validation of SLAMR. A. Bioanalyzer results testing the length and quality of the SLAMR full-length sense and antisense probes using for pull-down purification. **B**. Bar plots represent the relative gene expression of SLAMR and Actin control of RTqPCR results indicating and enrichment of SLAMR after the RNA isolation from PD samples compared to input and using two different sets of primers for SLAMR Multiple t Test, significance determined by Holm-Sidak method, *p-val<0.05, **p-val<0.01. **C.** Representative SDS-PAGE gel after silver staining that shows specific enrichments of proteins in the sense condition compared to the antisense. **D**. CamkIIα sequence coverage on LC-MS/MS showing the unique peptides counts of this protein. **E.** Vimentin sequence coverage on LC-MS/MS showing the unique peptides for this protein.

Supplementary Figure S6. A-F Cell body (top), dendrite (left) and kymographs of neurons transfected with MS2-SLAMR (A-B), MS2-SLAMRΔ92-289 (C-D) and MS2-SLAMRΔ898-1130 (E-F). Red arrows point to base of selected dendrite, green arrowheads point to anterograde tracks, red arrowheads point to retrograde tracks. **G-L.** Cell body (top), dendrite (left) and kymographs of neurons transfected with MS2-SLAMR (G-H), MS2-SLAMRΔ92-289 (I-J) and MS2-SLAMRΔ898-1130 (K-L) (green) and PSD95-mCherry(red). Red arrows point to base of the selected dendrite.

Supplementary Figure S7. SLAMR is not implicated in fear expression. A. Schematic mechanism of action of LNA-ASOs (Gapmers). **B.** D17Rik sequence showing the target sites for Gapmers 1, 2 and 3. **C.** Gapmers *in vitro* optimization for silencing SLAMR Dunnett's t-test * p<0.05, **p<0.005, ****p<0.0001. **D.** Gapmers *in vivo* optimization for silencing SLAMR by Bilateral stereotaxic infusions in dorsal CA1 at 72h (n=7). Student *t* test * p<0.05. **E.** The training curve did not show any significant differences in the expression of fear between groups for acquisition experiments. **F.** Training curve of fear expression representing the % of freezing time for memory consolidation experiment. Data does not indicate significant differences between groups. **G**. Training curve before silencing the expression of SLAMR in CA1 indicates no differences between groups at basal level. **H-K.** Adapted Allen Brain Atlas pictures shown the infusion position of the

subjects for negative control and SLAMR KD groups using black dots. **H.** Mice infused in CA1 for Acquisition experiment on CFC. **I**. Mice infused for consolidation experiment. **J.** Mice infused for Extinction and recall experiments. **K.** CA3 infusions of Gapmers for consolidation experiments. Scale bars = 1mm

Supplementary Figure S8. A. Photomicrograph represents the position of the infusion into dorsal hippocampus. Scale bar = 1mm. Images adapted from Allen mouse brain atlas represents the cannula placements indicated with black dots.

Supplementary Tables

Supplementary Table S1. List of significant genes from DEseq in dorsal CA1 after CFC training pertaining to Figure 1 and Supplementary Figure S1. Statistical analyses of all data related to Figures 1 and Supplementary Figures S1.

Supplementary Table S2. Statistical analyses of all data related to Figure 2.

Supplementary Table S3. Statistical analyses of all data related to Figure 3 and Supplementary Figure S3. Data revealing overexpression of KIF5C (KIF5C-OE) and SLAMR analysis by qPCR (Supplementary Table S3A).

Supplementary Table S4. Statistical analyses of all data related to Figure 4 and Supplementary Figure S4.

Supplementary Table S5. Statistical analyses of all data related to Figure 5 and Supplementary Figure S5.

Supplementary Table S6 RNA sequencing and LC-MS/MS data from SLAMR Biotin immunoprecipitation pertaining to Figure 6. Statistical analyses of all data related to Figure 6.

Supplementary Table S7. Statistical analyses of all data related to Figure 7 and Supplementary Figures S7

Supplementary Table S8. Statistical analyses of all data related to Figure 8.

Supplementary Table S9. Statistical analyses of all data related to Figure 9.

Supplementary Table S10. Primers used for all qRT-PCR experiments.

Supplementary Table S11. Sequence for sense and antisense in situ probes.

Supplementary movies

File Name: Movie S1
Description: Representative time-lapse movie of MCP alone lacking movement in a dendrite of a 15 DIV hippocampal neuron. Playback speed 30x real time. Total dendrite length=100μm. Related to Figure 2C.

File Name: Movie S2
Description: Representative time-lapse movie of *MS2-SLAMR* reporter mRNA granules moving in a dendrite of a 15 DIV hippocampal neuron. Playback speed 30x real time. Total dendrite length 100 μm. Related to Figure 2D.

File Name: Movie S3
Description: Representative time-lapse movie of MCP alone lacking movement in a dendrite of a 15 DIV hippocampal neuron. Playback speed 30x real time. Total dendrite length=100μm. Related to Supplementary Figure S2B.

File Name: Movie S4
Description: Representative time-lapse movie of *MS2-SLAMR* reporter mRNA granules moving in a dendrite of a 15 DIV hippocampal neuron. Playback speed 30x real time. Total dendrite length 100 μm. Related to Supplementary Figure S2C.

File Name: Movie S5
Description: Representative time-lapse movie of *MS2-SLAMR* reporter mRNA granules (green) moving and dendritic spines labeled with PSD95-mCherry (red) in a dendrite of a 15 DIV hippocampal neuron. Playback speed 30x real time. Total dendrite length 100 μm. Related to Supplementary Figure S2D.

File Name: Movie S6
Description: Representative time-lapse movie of *MS2-SLAMR* reporter mRNA granules (green) moving and dendritic spines labeled with PSD95-mCherry (red) in a dendrite of a 15 DIV hippocampal neuron. Playback speed 30x real time. Total dendrite length 100 μm. Related to Supplementary Figure S2E.

File Name: Movie S7
Description: Representative time-lapse movie of *MS2-SLAMR:MCP-RFP* reporter mRNA granules (red) moving in a 15 DIV hippocampal neuron transfected with Scr-shRNA (GFP). Playback speed 30x real time. Total dendrite length 100 μm. Related to Supplementary Figure S3A.

File Name: Movie S8
Description: Representative time-lapse movie of *MS2-SLAMR:MCP-RFP* reporter mRNA granules (red) moving in a 15 DIV hippocampal neuron transfected with Scr-shRNA (GFP). Playback speed 30x real time. Total dendrite length 100 μm. Related to Supplementary Figure S3B.

File Name: Movie S9
Description: Representative time-lapse movie of *MS2-SLAMR:MCP-RFP* reporter mRNA granules (red) moving in a 15 DIV hippocampal neuron transfected with KIF5C-shRNA (GFP). Playback speed 30x real time. Total dendrite length 100 µm. Related to Supplementary Figure S3C.

File Name: Movie S10
Description: Representative time-lapse movie of *MS2-SLAMR:MCP-RFP* reporter mRNA granules (red) moving in a 15 DIV hippocampal neuron transfected with KIF5C-shRNA (GFP). Playback speed 30x real time. Total dendrite length 100 µm. Related to Supplementary Figure S3D.

File Name: Movie S11
Description: Representative time-lapse movie of *MS2-SLAMR:MCP-RFP* MCP lacking movement in a dendrite of a 19 DIV hippocampal neuron, taken 3 minutes after spine stimulation in a nonresponsive spine. Stimulated spine positioned in the middle. Playback speed 30x real time. Total dendrite length=50µm. Related to Supplementary Figure S3H.

File Name: Movie S12
Description: Representative time-lapse movie of *MS2-SLAMR:MCP-RFP* MCP showing movement toward the spine in a dendrite of a 19 DIV hippocampal neuron, taken 3 minutes after spine stimulation in a responsive spine. Stimulated spine positioned in the middle. Playback speed 30x real time. Total dendrite length=50µm. Related to Supplementary Figure S3I.

File Name: Movie S13
Description: Representative time-lapse movie of *MS2-SLAMR* reporter mRNA granules moving in a dendrite of a 15 DIV hippocampal neuron. Playback speed 30x real time. Total dendrite length 100 µm. Related to Supplementary Figure S6A.

File Name: Movie S14
Description: Representative time-lapse movie of *MS2-SLAMR* reporter mRNA granules moving in a dendrite of a 15 DIV hippocampal neuron. Playback speed 30x real time. Total dendrite length 100 µm. Related to Supplementary Figure S6B.

File Name: Movie S15
Description: Representative time-lapse movie of *MS2-SLAMRΔ92-289* reporter mRNA granules moving in a dendrite of a 15 DIV hippocampal neuron. Playback speed 30x real time. Total dendrite length 100 µm. Related to Supplementary Figure S6C.

File Name: Movie S16
Description: Representative time-lapse movie of *MS2-SLAMRΔ92-289* reporter mRNA granules moving in a dendrite of a 15 DIV hippocampal neuron. Playback speed 30x real time. Total dendrite length 100 µm. Related to Supplementary Figure S6D.

File Name: Movie S17
Description: Representative time-lapse movie of *MS2-SLAMRΔ898-1130* reporter mRNA granules moving in a dendrite of a 15 DIV hippocampal neuron. Playback speed 30x real time. Total dendrite length 100 µm. Related to Supplementary Figure S7E.

File Name: Movie S18
Description: Representative time-lapse movie of *MS2-SLAMRΔ898-1130* reporter mRNA granules moving in a dendrite of a 15 DIV hippocampal neuron. Playback speed 30x real time. Total dendrite length 100 µm. Related to Supplementary Figure S6F.

File Name: Movie S19
Description: Representative time-lapse movie of *MS2-SLAMR* reporter mRNA granules (green) moving and dendritic spines labeled with PSD95-mCherry (red) in a dendrite of a 15 DIV hippocampal neuron. Playback speed 30x real time. Total dendrite length 100 µm. Related to Supplementary Figure S6G.

File Name: Movie S20
Description: Representative time-lapse movie of *MS2-SLAMR* reporter mRNA granules (green) moving and dendritic spines labeled with PSD95-mCherry (red) in a dendrite of a 15 DIV hippocampal neuron. Playback speed 30x real time. Total dendrite length 100 µm. Related to Supplementary. Figure S6H.

File Name: Movie S21
Description: Representative time-lapse movie of *MS2-SLAMRΔ92-289* reporter mRNA granules (green) moving and dendritic spines labeled with PSD95-mCherry (red) in a dendrite of a 15 DIV hippocampal neuron. Playback speed 30x real time. Total dendrite length 100 µm. Related to Supplementary Figure S6I.

File Name: Movie S22
Description: Representative time-lapse movie of *MS2-SLAMRΔ92-289* reporter mRNA granules (green) moving and dendritic spines labeled with PSD95-mCherry (red) in a dendrite of a 15 DIV hippocampal neuron. Playback speed 30x real time. Total dendrite length 100 µm. Related to Supplementary Figure S6J.

File Name: Movie S23
Description: Representative time-lapse movie of *MS2-SLAMRΔ898-1130* reporter mRNA granules (green) moving and dendritic spines labeled with PSD95-mCherry (red) in a dendrite of a 15 DIV hippocampal neuron. Playback speed 30x real time. Total dendrite length 100 µm. Related to Supplementary Figure S6K.

File Name: Movie S24
Description: Representative time-lapse movie of *MS2-SLAMRΔ898-1130* reporter mRNA granules (green) moving and dendritic spines labeled with PSD95-mCherry (red) in a dendrite of a 15 DIV hippocampal neuron. Playback speed 30x real time. Total dendrite length 100 µm. Related to Supplementary Figure S6L.

SLAMR, a synaptically targeted lncRNA, facilitates the consolidation of contextual fear memory

Isabel Espadas[1#], Jenna Wingfield[1#], Eddie Grinman[1], Ilika Ghosh[2], Kaushik Chanda[1], Yoshihisa Nakahata[2], Karl Bauer[3], Bindu Raveendra[1], Michael Kiebler[3], Ryohei Yasuda[2], Vidhya Rangaraju[2], Sathyanarayanan Puthanveettil[1*]

8 Supplementary Figures (S1-S8)

Supplementary Figure S1

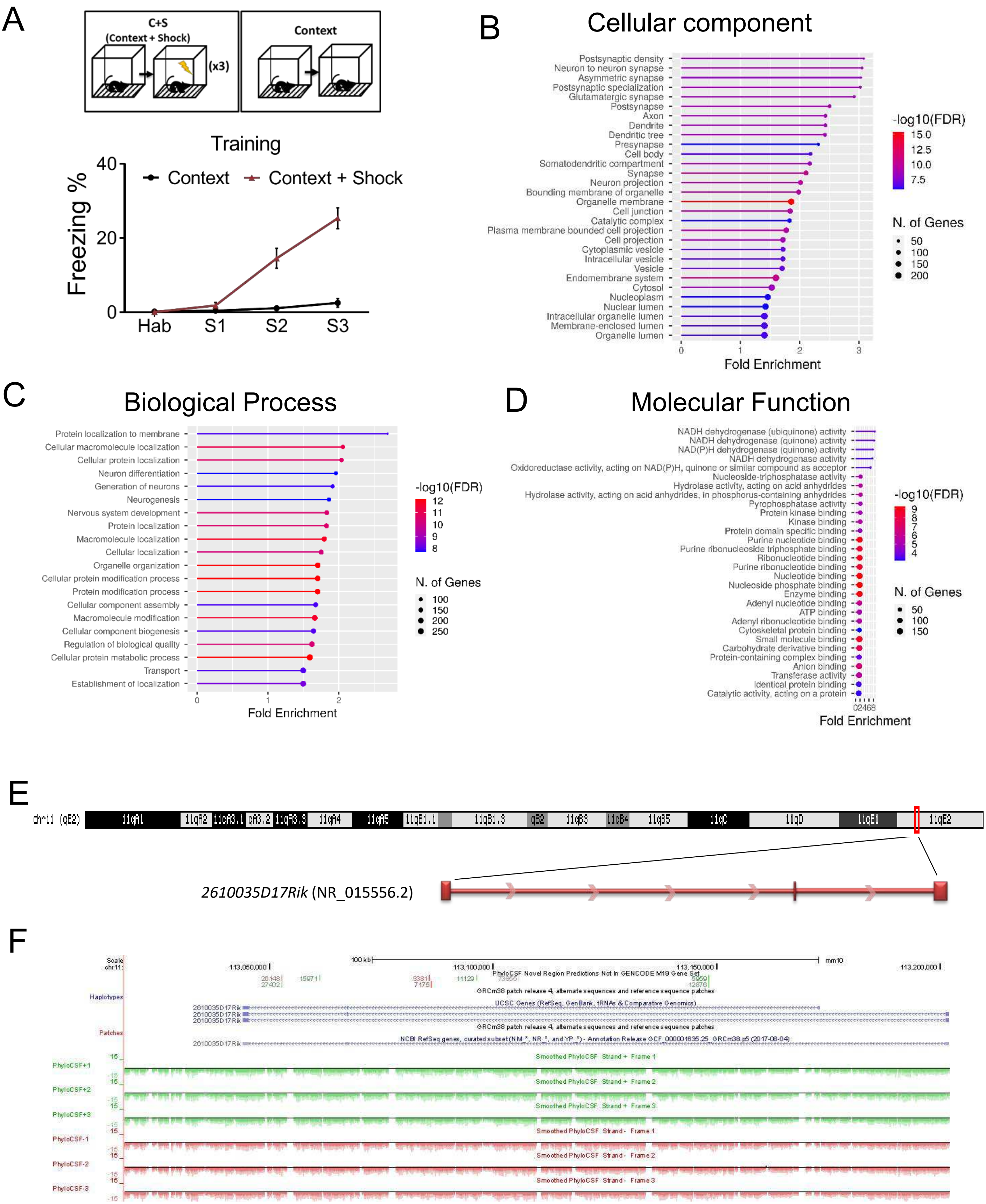

G

CPAT Coding Potential Assessment Tool

Result for species name : mm9 with job ID :1598537962							
Data ID	Sequence Name	RNA Size	ORF Size	Ficket Score	Hexamer Score	Coding Probability	Coding Label
0	NR_015556.2	1848	237	0.8075	0.137375310793	0.1663786723022	no

Supplementary Figure S1

H

Mouse (*Mus musculus*)

Chr11

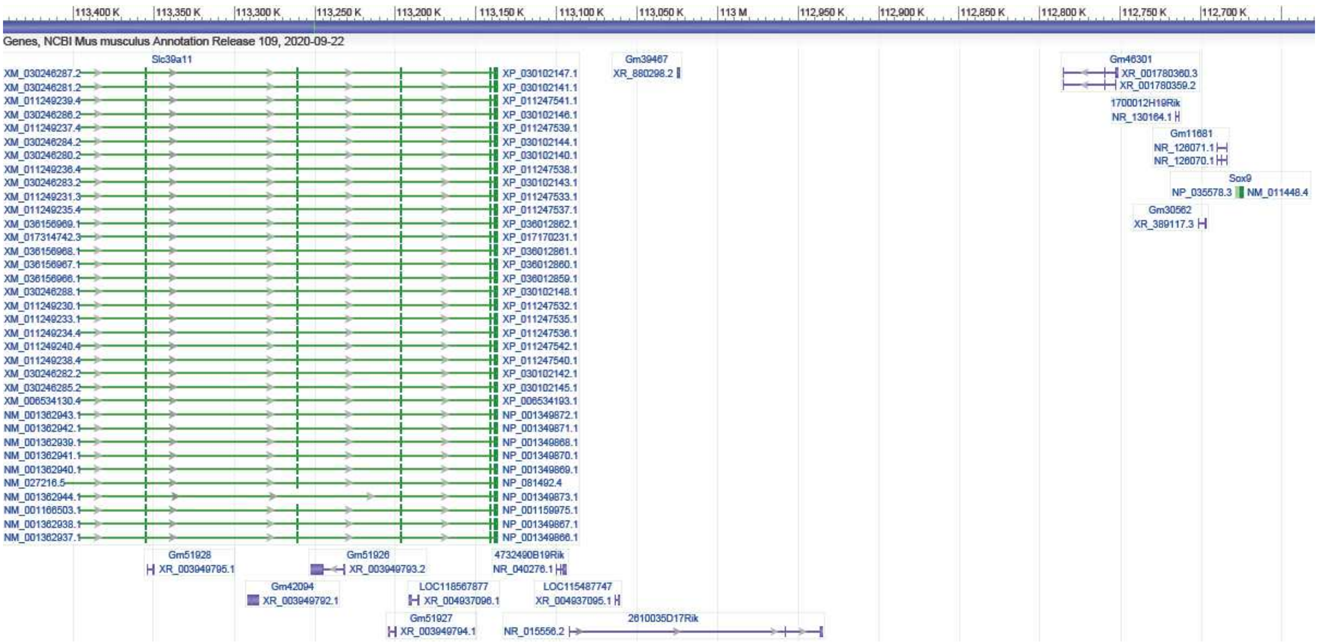

I

Human (*Homo sapiens*)

Chr17

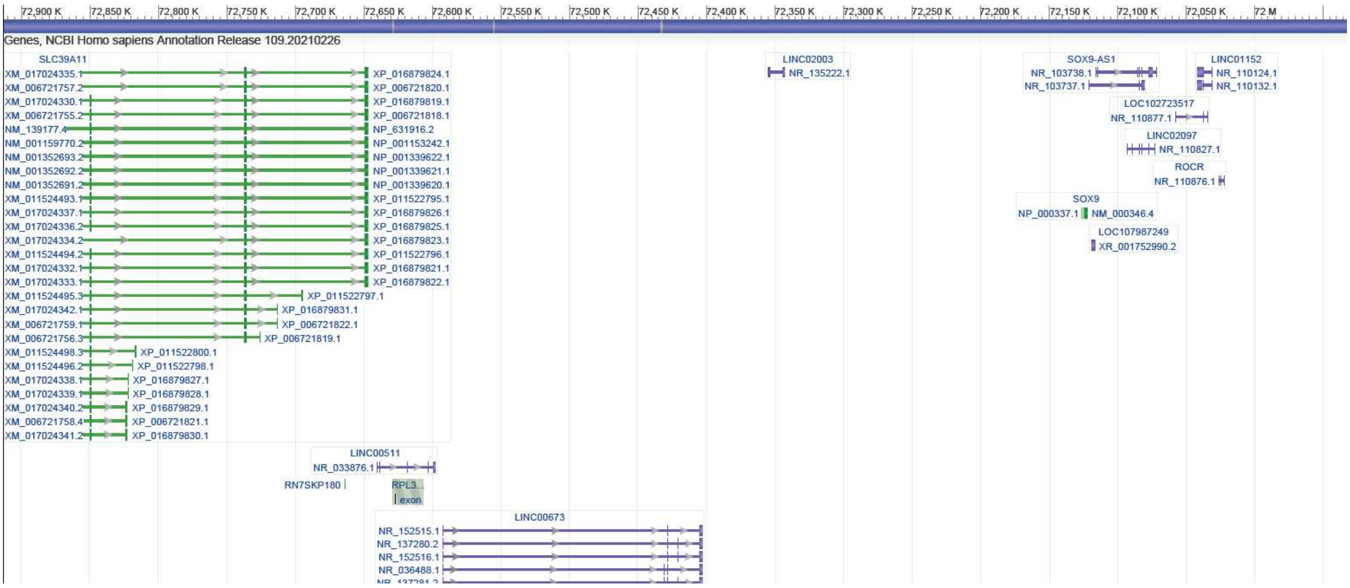

J

Zebra Fish (*Danio rerio*)

Chr3

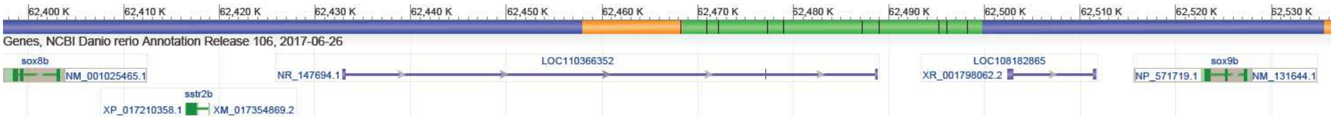

Supplementary Figure S1

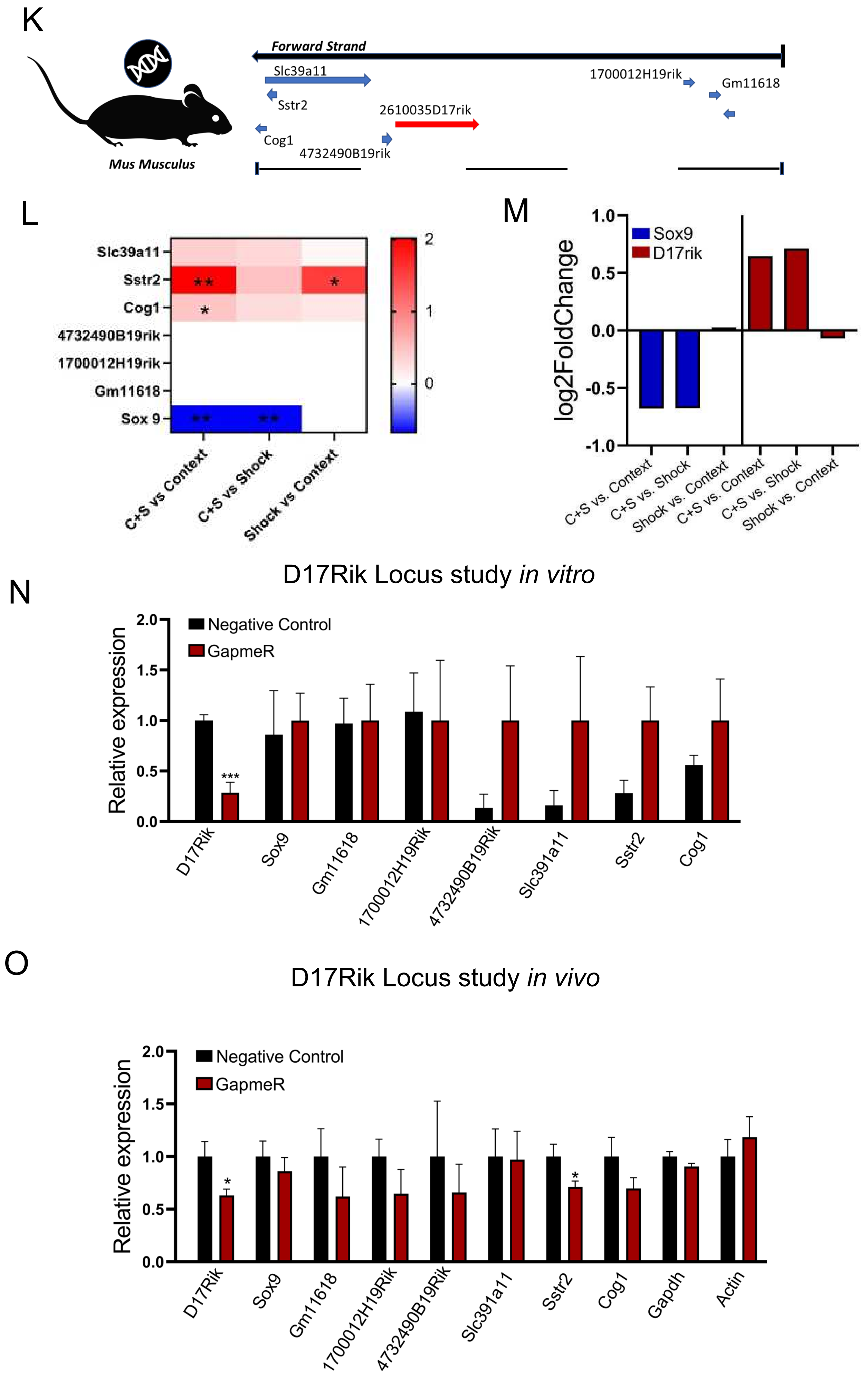

P D17Rik distribution in hippocampus

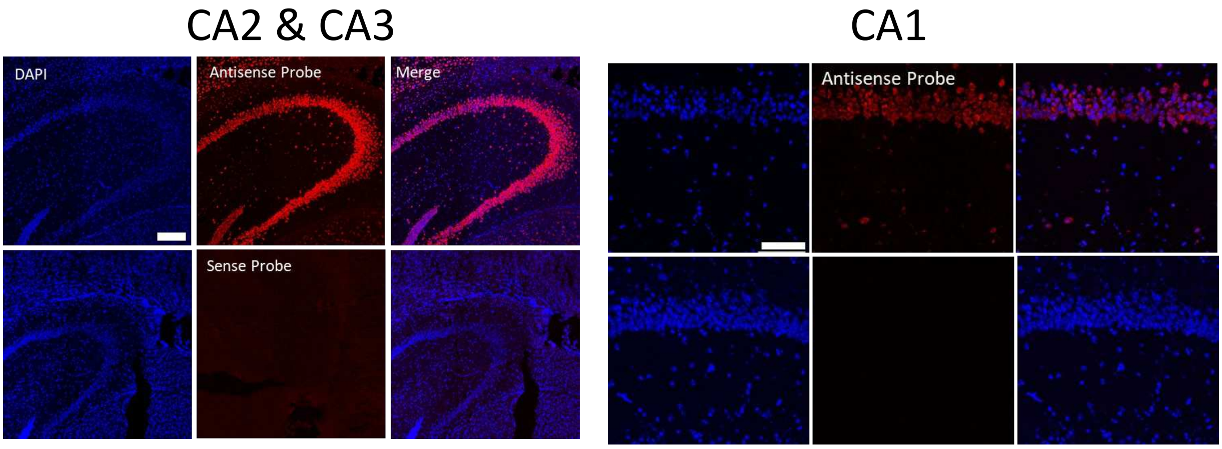

Q Fractionation

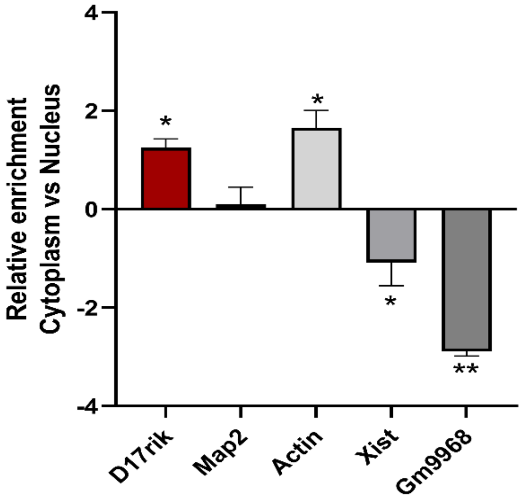

Supplementary Figure S2

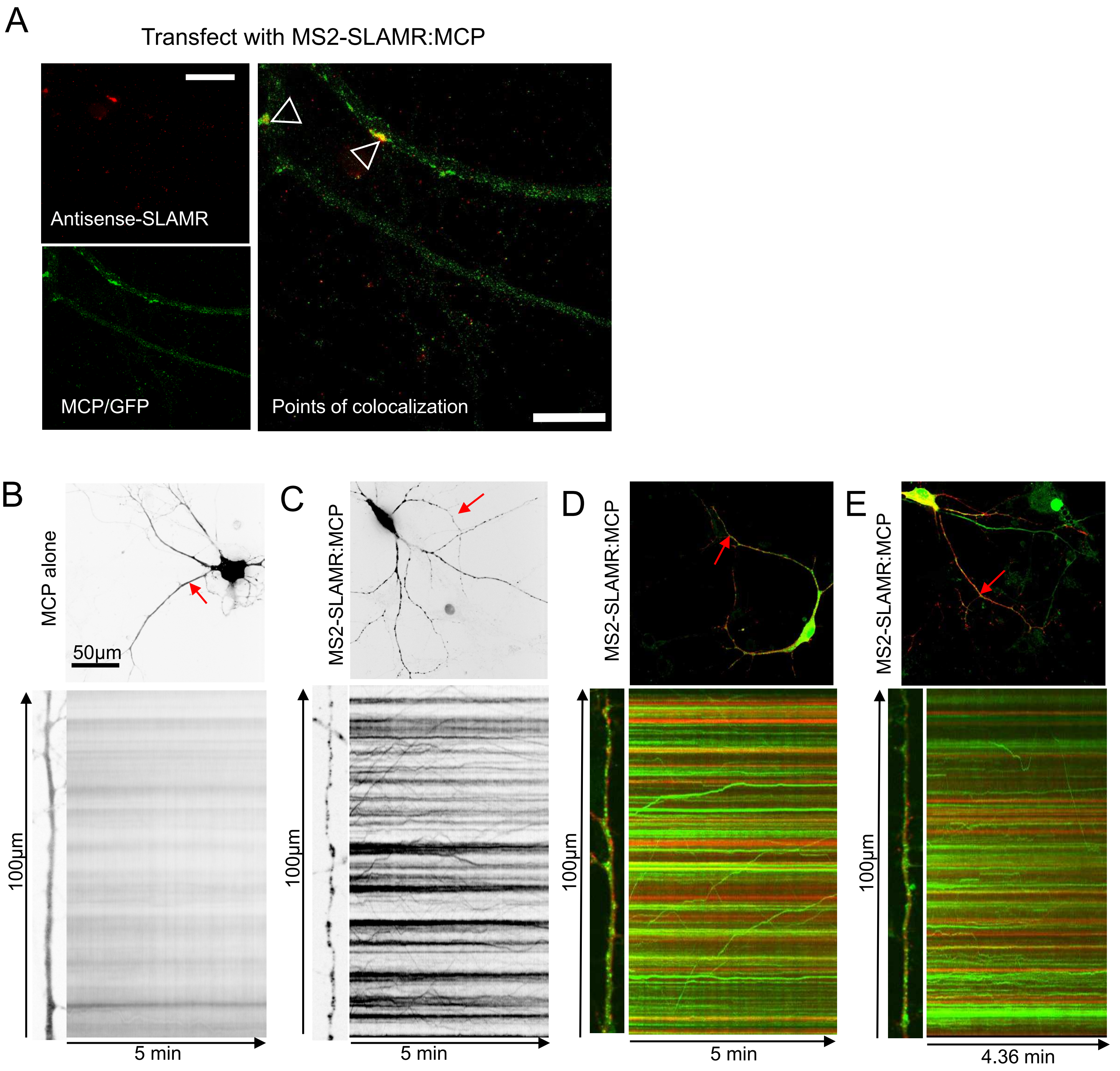

Supplementary Figure S3

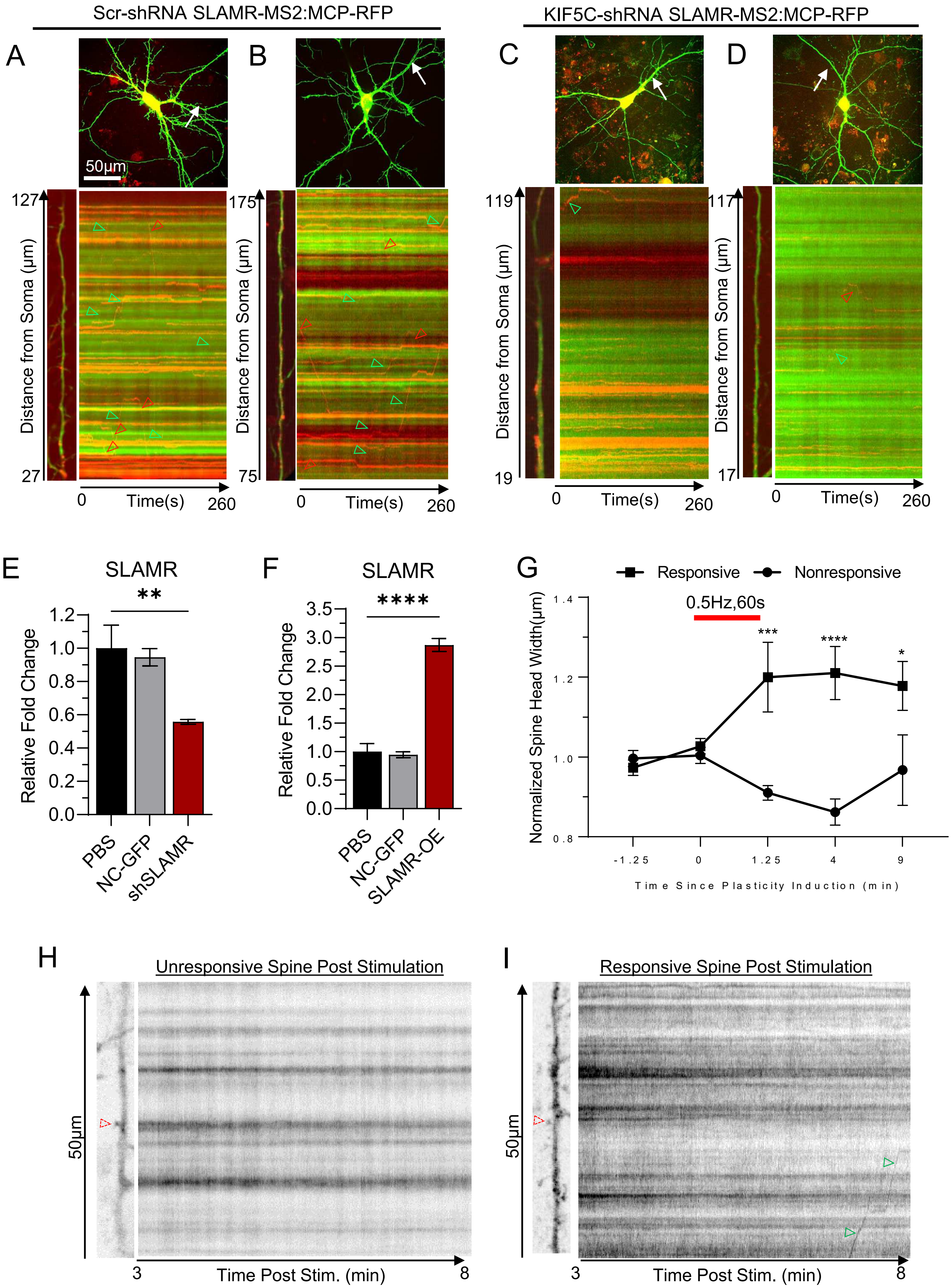

Supplementary Figure S4

Validation of Synaptoneurosome Isolation

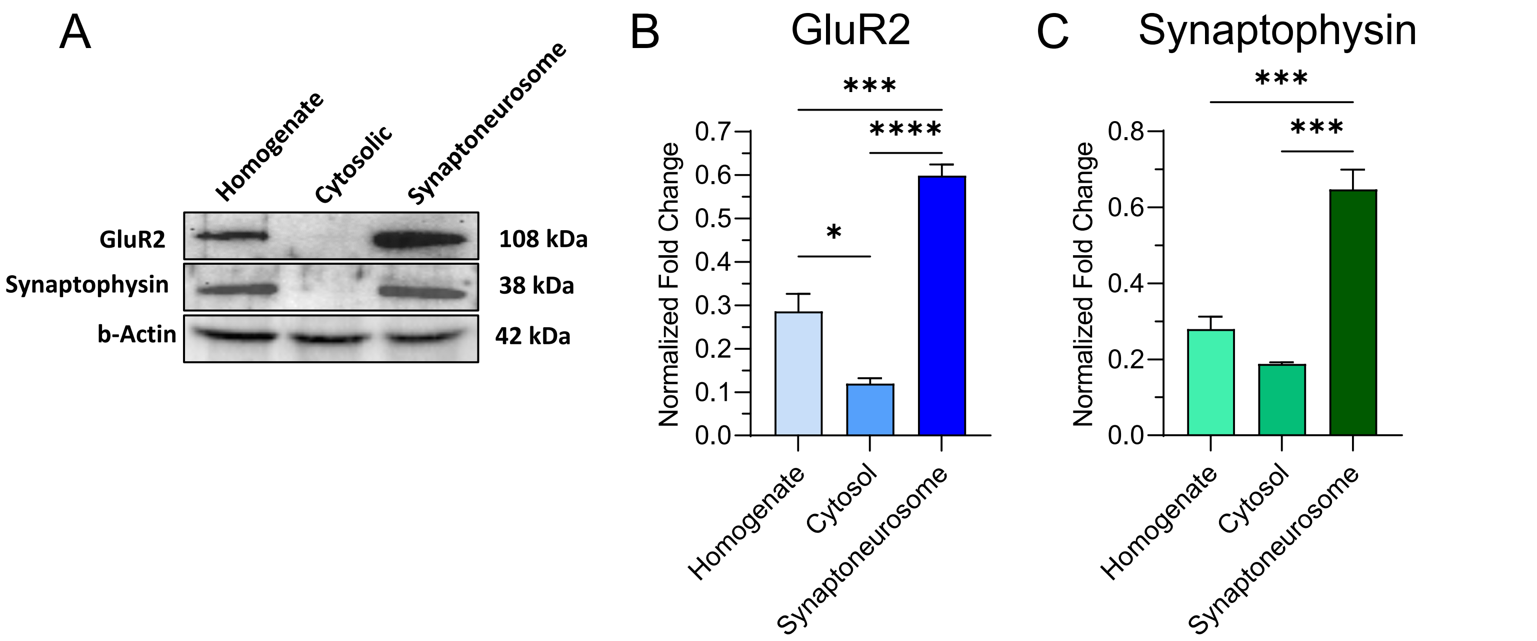

Supplementary Figure S5

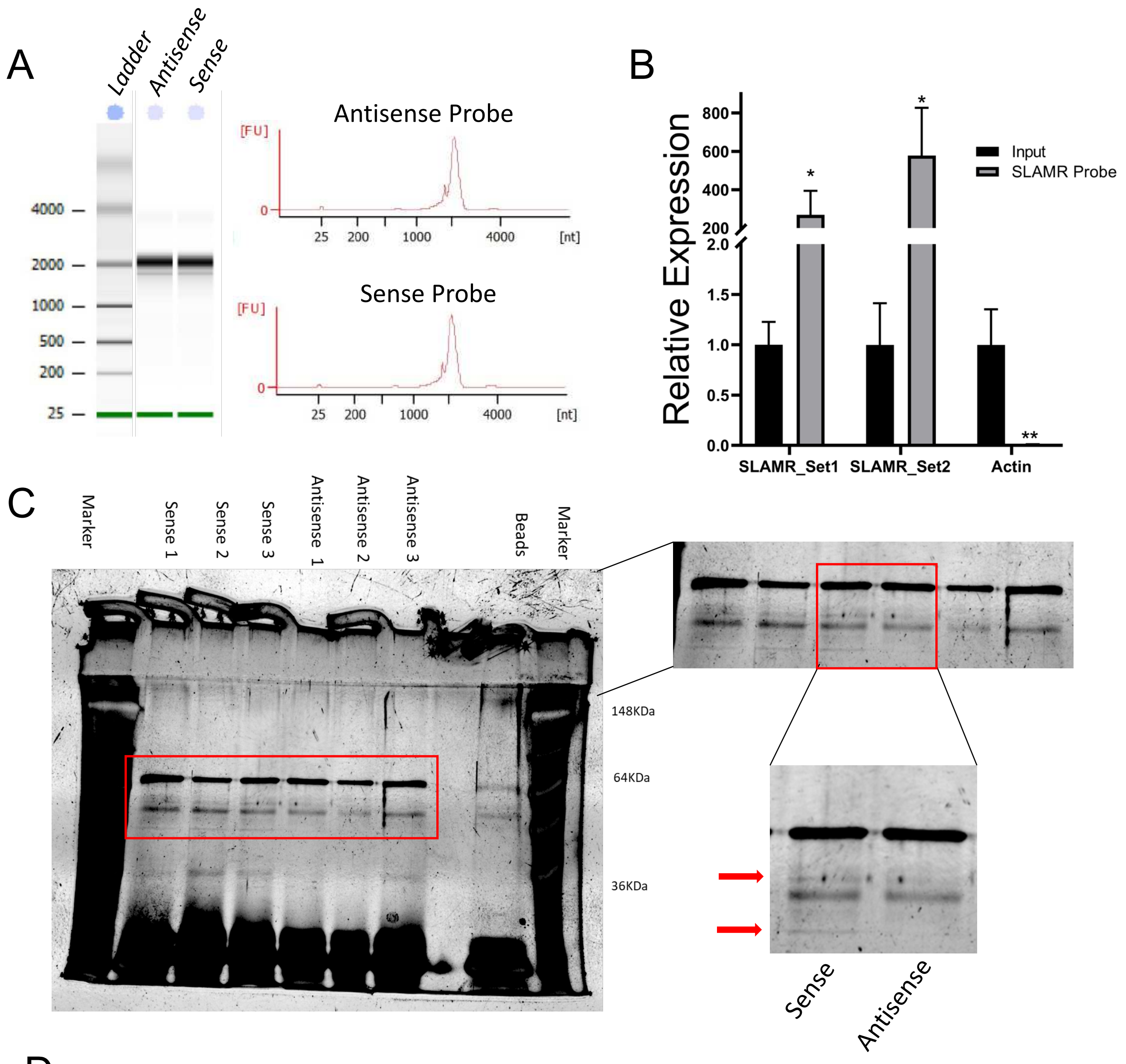

D CamKIIα sequence coverage

Sequence Coverage	Protein	Accession	Prob	%S...	#Pep
	Calcium/calmodulin-dependent protein k...	sp|P11798|...	100%	0.01...	5

sp|P11798|KCC2A_MOUSE (100%), 54.115,7 Da
Calcium/calmodulin-dependent protein kinase type II subunit alpha OS=Mus musculus OX=10090 GN=Camk2a PE=1 SV=2
5 exclusive unique peptides, 5 exclusive unique spectra, 5 total spectra, 61/478 amino acids (13% coverage)

MATITCTRFT EEYQLFEELG KGAFSVVRRC VKVLAGQEYA AKIINTKKLS
ARDHQKLERE ARICRLLKHP NIVRLHDSIS EEGHHYLIFD LVTGGELFED
IVAREYYSEA DASHCIQQIL EAVLHCHQMG VVHRDLKPEN LLLASKLKGA
AVKLADFGLA IEVEGEQQAW FGFAGTPGYL SPEVLRKDPY GKPVDLWACG
VILYILLVGY PPFWDEDQHR LYQQIKAGAY DFPSPEWDTV TPEAKDLINK
MLTINPSKRI TAAEALKHPW ISHRSTVASC MHRQETVDCL KKFNARRKLK
GAILTTMLAT RNFSGGKSGG NKKNDGVKES SESTNTTIED EDTKVRKQEI
IKVTEQLIEA ISNGDFESYT KMCDPGMTAF EPEALGNLVE GLDFHRFYFE
NLWSRNSKPV HTTILNPHIH LMGDESACIA YIRITQYLDA GGIPRTAQSE
ETRVWHRRDG KWQIVHFHRS GAPSVLPH

E Vimentin sequence coverage

Sequence Coverage	Protein	Accession	Category	Bio Sample	MS/MS Sample	Prob	%S...	#Pep
	Vimentin OS=Mus mu...	sp|P20152...	Sense	MS219051_SP...		100%	0,11%	20

sp|P20152|VIME_MOUSE (100%), 53.688,8 Da
Vimentin OS=Mus musculus OX=10090 GN=Vim PE=1 SV=3
20 exclusive unique peptides, 21 exclusive unique spectra, 30 total spectra, 285/466 amino acids (61% coverage)

MSTRSVSSSS YRRMFGGSGT SSRPSSNRSY VTTSTRTYSL GSALRPSTSR
SLYSSSPGGA YVTRSSAVRL RSSVPGVRLL QDSVDFSLAD AINTEFKNTR
TNEKVELQEL NDRFANYIDK VRFLEQQNKI LLAELEQLKG QGKSRLGDLY
EEEMRELRRQ VDQLTNDKAR VEVERDNLAE DIMRLREKLQ EEMLQREEAE
STLQSFRQDV DNASLARLDL ERKVESLQEE IAFLKKLHDE EIQELQAQIQ
EQHVQIDVDV SKPDLTAALR DVRQQYESVA AKNLQEAEEW YKSKFADLSE
AANRNNDALR QAKQESNEYR RQVQSLTCEV DALKGTNESL ERQMREMEEN
FALEAANYQD TIGRLQDEIQ NMKEEMARHL REYQDLLNVK MALDIEIATY
RKLLEGEESR ISLPLPTFSS LNLRETNLES LPLVDTHSKR TLLIKTVETR
DGQVINETSQ HHDDLE

Supplementary Figure S6

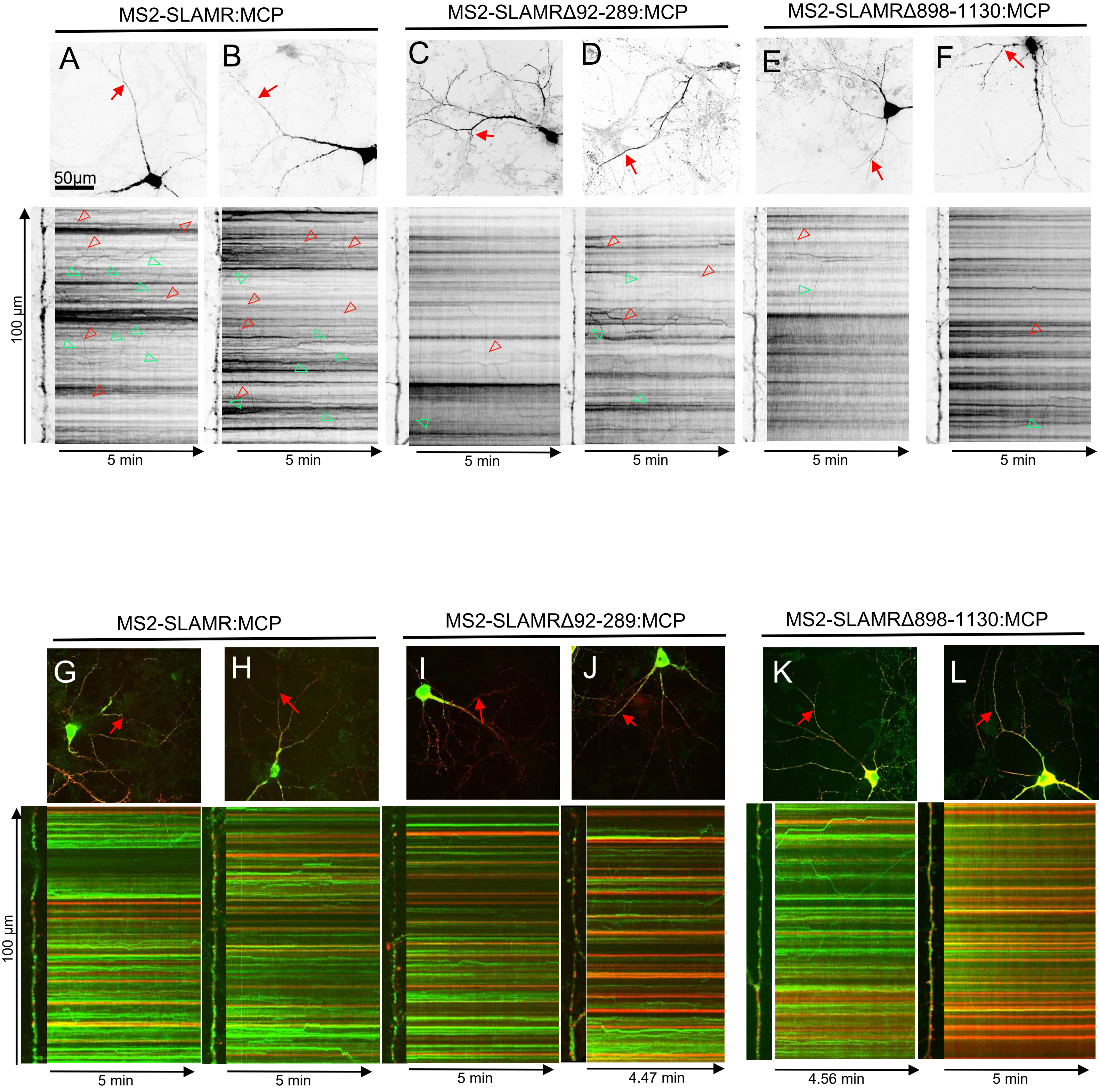

Supplementary Figure S7

A

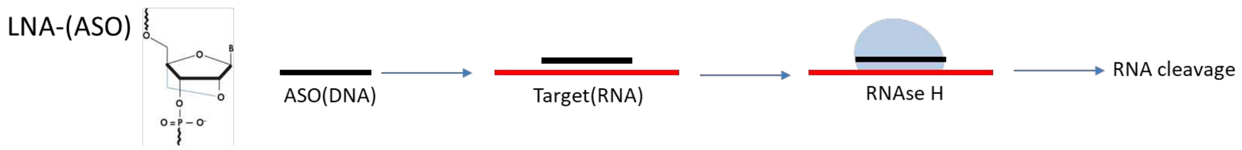

B

>NR_015556.2 Mus musculus RIKEN cDNA 2610035D17 gene (2610035D17Rik), long non-coding RNA

```
AGAGCTCAGTGGTGGCGCAGAAAGTGTTCACTTCTTCTGTTTCGGGCTGTACGCTGTGGTTCTGCGCCTC
CAGGGCTTTGCTGCCTGGCGAGCGTCCTCCCTGTACCACAGGATCCTTGCCCGACTACAAACCCGAGGCT
TCTATTAGGATAGTCTGGATTCGCCATAGGGGTTTCTCCTAGCGCGGATTGTGTGTTTGCGTTGGCGATG
CTCATAGAAGAAAGGCGCAGAGCTGTACGAGGAGGCGGGATGCACGGGTCAGAGCCCTCGGTGGCCTCCG
CCCGCTGCTCTTGGCGCCGGTCCGCTCCTGGAGATAGCGCGGCAGCCGCCTGGGAGGCGAAGCGCGTAAT
CTCCGCGCTACCCACGCGGGGCCGCGGCGTGGTGGCCGCACTTTGGCTTTGGGCGCTGGATGGACAGTGG
GGTAAAGGGTTGGGGTTCCCCTAACGTGGATTACTTGGTGCTTATCAACTGGATTTAGAAGACTCTCGAC
CTGGTCTCCATCATCCGTGGGTTGTCTTTCCATCTCCCAGCCGCTCTTTCACCTGGAGGTGAAACCAGAG
CTTCTGCCTGCATGGAACAAGAGCAAGAGAAAGGCCGAGGAGACTGACCTGCTTATCCATGCTACTTATC
AGGAACTGTGGAACCATGGAGAACATGACCCTGGCCCTCCCTTCAGGATCTGCCTGAGATCTGGCTGCCT
CTTGAGACACTAGAGCCCTGGATTCAATTGCGGAACATTATGTTGGAGAATATAAACACTTCTAGACTGT
GCCGGCATTTGGGAAGGAATAGTCTGTTTTGACTTGAATGAACAAAGACATACGTCTCTGGGCCTGGGGA
AGGCTTACCAGCTCAGCTCATCTACTGTCTGGTGGCAAACATGTCCTGGGTACTGCCACTACCCAGGACA
CTCTTCCTCACTCTGTCCCTGTCCAGTTACCGCACCCTTCCCTGTCTGACCCCGCCTCCTGTGCCCACTC
GCCTCTGGACCCAGAGATTCTTCTTGTTTGCTCTATCATCCTTGAGCCGGATACAGGGTGAATAGTTAAC
CACACTTGGGTTGAATGGGATCTTGATTCTTATTTCTGTTCCTTGGAAAGAGGGGTCAGACTCGGTGCGT
GGCACCCGGCCTGGAAGCAGCACAAGACTCCAGAGTGATACATGATCTGCCTGGATGGTCCCAGGGGCTC
TTTGGAGGTCAGAGCAAGAGGAGCAGCCTGAGCAGGGGGGCTGAGGAGGGCCGGCACTGGATCACACGCA
GGCGTATGTCTGGATAGGCAGAGAGGAACCCACTTTTCATTCCCTCTTCTGTGACACAAATGCAAGGAAC
TTTGAAGACACTCTTCGGAGGTTGCAATCGGAGATCAGCTTATCCAAGGGCTTCATGTCTGAGCTCTGTT
GGTCGTGCCTCCTGAGGACCTCTTTCCTCTACTGTATGCTGATGACACACCCATCCGATGGGAGCTGGAG
CCTTGTCTGGTCCTTTCCTCTATGACCCCACGTGTACAGTTGGAAATCCTTCCTAATCATGCCTTGATTC
CCCCTTTTCGACTACACTCAAAGATGTTTGAGCTGTTTTATTTGCAAAATGGATGGACAGTTTGTTTATA
TAAAACAATCCCAGCTTCCTTGTGCTCTATCTGGTGTGTTTGTATGGCTCTTCAGTGACTTTAAAGTGGA
GCATTGACCTTGGCCTCTTTGATTCTCCATGGTGGAATAGAGAAGACCTCTTGAGGATGCTTAATGTTGC
TGAGATCTTAAGAAGGCTTGCAGACTTCCTCTTCCTCCCTCCTTCTCCCACCTCCTCCTTCCCTAAGCAG
CCTCTGCATTGTATAAATTCCTTTATAT
```

Gapmer_1 (CCTGTCCAGTTACCGC) **Gapmer_2** (TGTTTGCTCTATCATC) **Gapmer_3** (ACTACACTCAAAGATG)

C

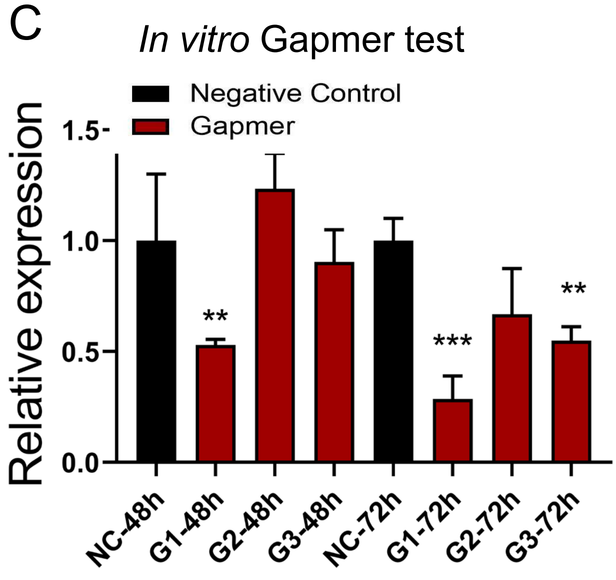

D

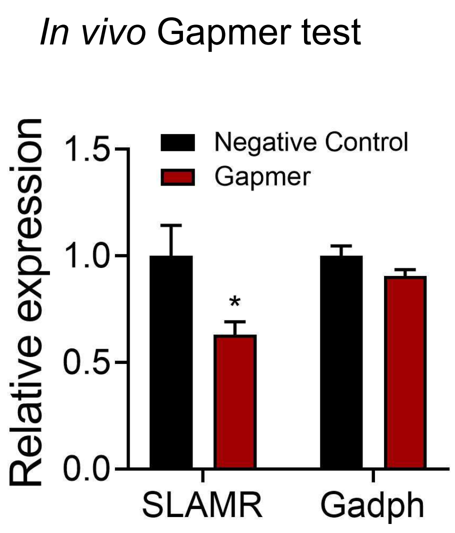

Supplementary Figure S7

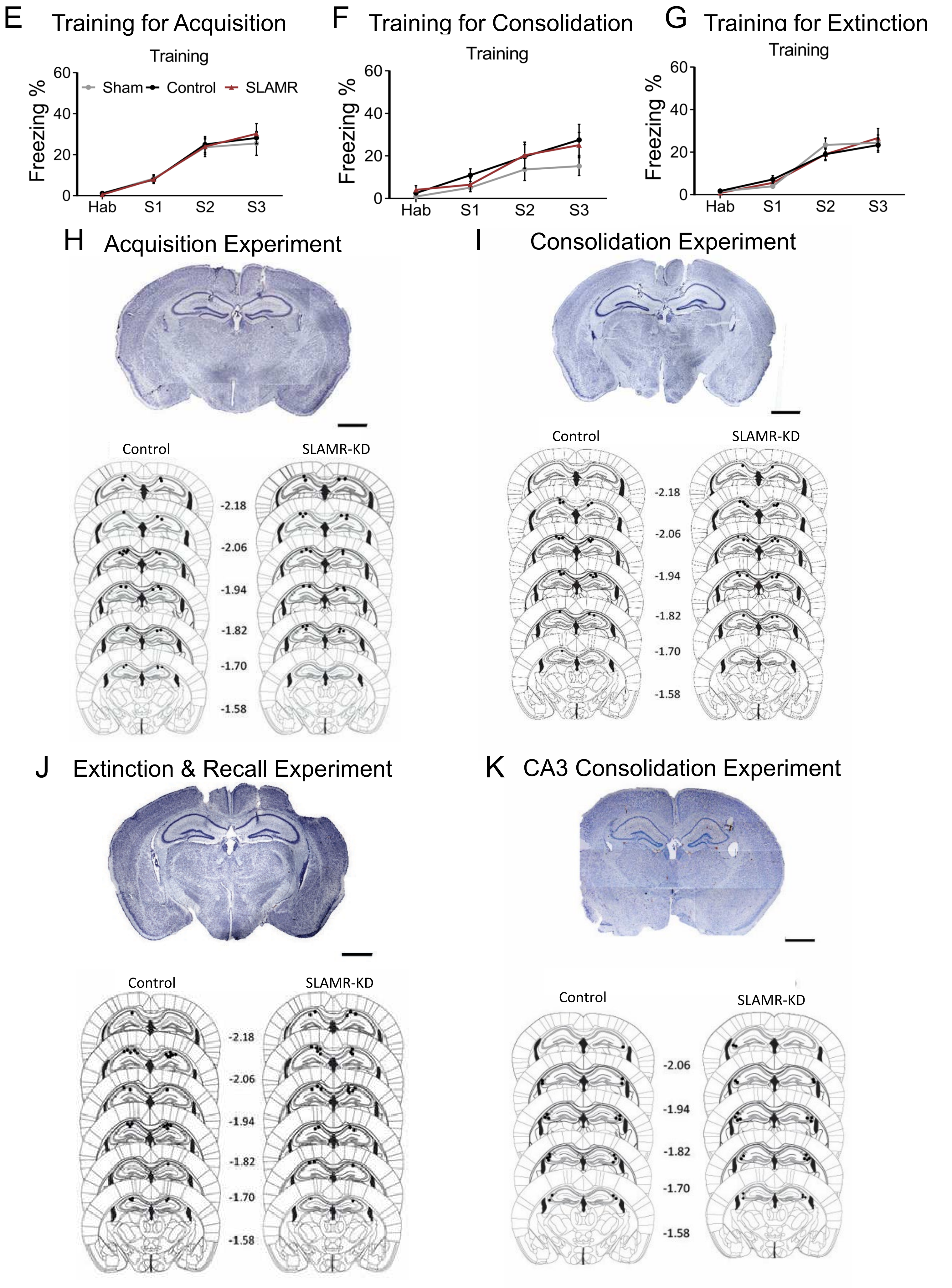

Supplementary Figure S8

A Morris Water Maze Experiment

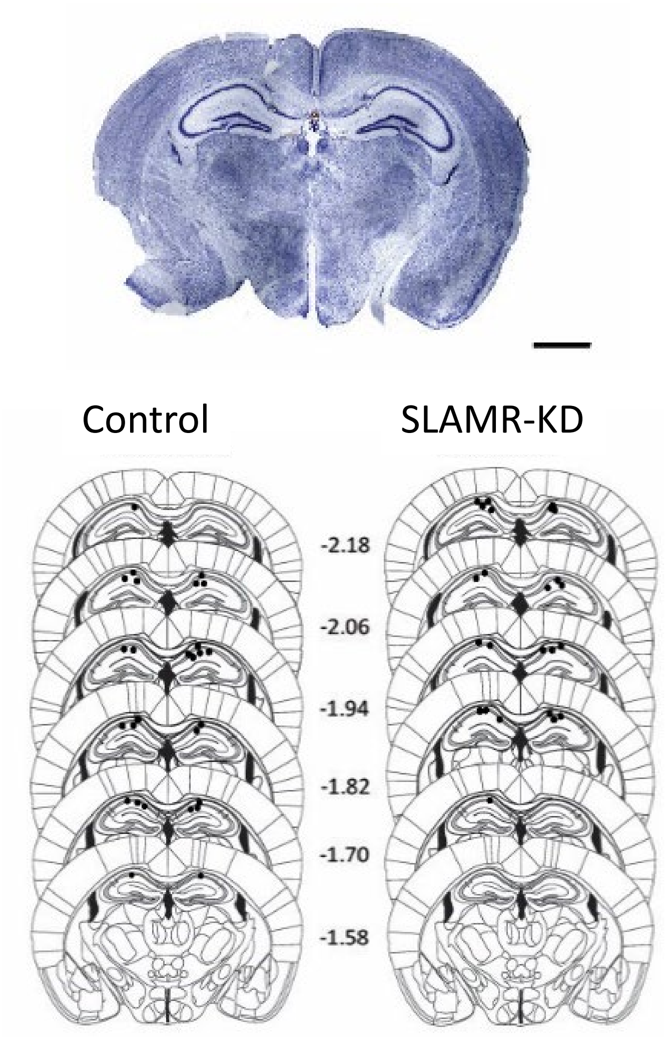

Supplementary Files

This is a list of supplementary files associated with this preprint. Click to download.

- MovieS14.avi
- MovieS10.avi
- MovieS6.avi
- MovieS5.avi
- MovieS11.avi
- MovieS16.avi
- MovieS17.avi
- MovieS18.avi
- MovieS3.avi
- MovieS1.avi
- MovieS9.avi
- MovieS23.avi
- MovieS12.avi
- MovieS15.avi
- MovieS2.avi
- MovieS21.avi
- MovieS13.avi
- MovieS4.avi
- MovieS22.avi
- MovieS20.avi
- MovieS24.avi
- MovieS7.avi
- MovieS8.avi
- MovieS19.avi
